# Butyrate and Dietary Soluble Fiber Improve Neuroinflammation Associated With Aging in Mice

**DOI:** 10.3389/fimmu.2018.01832

**Published:** 2018-08-14

**Authors:** Stephanie M. Matt, Jacob M. Allen, Marcus A. Lawson, Lucy J. Mailing, Jeffrey A. Woods, Rodney W. Johnson

**Affiliations:** ^1^Neuroscience Program, University of Illinois at Urbana-Champaign, Urbana, IL, United States; ^2^Department of Animal Sciences, University of Illinois at Urbana-Champaign, Urbana, IL, United States; ^3^Department of Kinesiology and Community Health, University of Illinois at Urbana-Champaign, Urbana, IL, United States; ^4^Division of Nutritional Sciences, University of Illinois at Urbana-Champaign, Urbana, IL, United States; ^5^Integrative Immunology and Behavior Program, University of Illinois at Urbana-Champaign, Urbana, IL, United States

**Keywords:** microglia, butyrate, aging, neuroinflammation, epigenetics, microbiome, fiber diet

## Abstract

Aging results in chronic systemic inflammation that can alter neuroinflammation of the brain. Specifically, microglia shift to a pro-inflammatory phenotype predisposing them to hyperactivation upon stimulation by peripheral immune signals. It is proposed that certain nutrients can delay brain aging by preventing or reversing microglial hyperactivation. Butyrate, a short-chain fatty acid (SCFA) produced primarily by bacterial fermentation of fiber in the colon, has been extensively studied pharmacologically as a histone deacetylase inhibitor and serves as an attractive therapeutic candidate, as butyrate has also been shown to be anti-inflammatory and improve memory in animal models. In this study, we demonstrate that butyrate can attenuate pro-inflammatory cytokine expression in microglia in aged mice. It is still not fully understood, however, if an increase in butyrate-producing bacteria in the gut as a consequence of a diet high in soluble fiber could affect microglial activation during aging. Adult and aged mice were fed either a 1% cellulose (low fiber) or 5% inulin (high fiber) diet for 4 weeks. Findings indicate that mice fed inulin had an altered gut microbiome and increased butyrate, acetate, and total SCFA production. In addition, histological scoring of the distal colon demonstrated that aged animals on the low fiber diet had increased inflammatory infiltrate that was significantly reduced in animals consuming the high fiber diet. Furthermore, gene expression of inflammatory markers, epigenetic regulators, and the microglial sensory apparatus (i.e., the sensome) were altered by both diet and age, with aged animals exhibiting a more anti-inflammatory microglial profile on the high fiber diet. Taken together, high fiber supplementation in aging is a non-invasive strategy to increase butyrate levels, and these data suggest that an increase in butyrate through added soluble fiber such as inulin could counterbalance the age-related microbiota dysbiosis, potentially leading to neurological benefits.

## Introduction

During healthy aging, there is a disruption in the communication and balance between the brain and immune system. Microglia shift to a pro-inflammatory phenotype that makes them hypersensitive to signals from the peripheral immune system (1, 2). The precise mechanisms during aging that are responsible for this detrimental transition is not known, but overproduction of the pro-inflammatory cytokine interleukin (IL)-1β has been shown to play a role. In a recent study, we demonstrated that aged mice had decreased methylation of the IL-1β gene promoter in primary microglia basally or following systemic lipopolysaccharide (LPS) that is associated with increased IL-1β mRNA (3). This is critical because overproduction of IL-1β can cause cognitive dysfunction in rodent models (4) and increased IL-1β in aged individuals is associated with risk of neurodegenerative diseases such as Alzheimer’s (5).

It is proposed that certain nutrients can manipulate the epigenome to delay brain aging by preventing or reversing microglial aging, potentially through DNA methylation, histone modifications, and their interactions (6). Butyrate, a short-chain fatty acid (SCFA) produced by bacterial fermentation of fiber in the colon, has been extensively studied for its beneficial effects through free fatty acid receptor signaling and metabolic regulation, but also pharmacologically as a histone deacetylase (HDAC) inhibitor. HDAC inhibitors have become an attractive therapeutic candidate due to the ability to increase histone acetylation and promote the expression of neurotrophic and anti-inflammatory genes (7, 8). Although most studies using butyrate have focused on its effects on histone acetylation, there is also evidence that butyrate can affect DNA methylation (9–11).

Sodium butyrate (NaB), the sodium salt form of butyrate, is most commonly used in pharmacological studies and has been shown to decrease pro-inflammatory cytokines of microglia *in vitro* as well as improve learning and memory in mouse models of neurodegenerative diseases when injected intraperitoneally (i.p.) (12, 13). It has also recently been demonstrated that NaB triggers elongation of microglial processes and can abolish LPS-induced depressive-like behaviors through decreasing microglial activation (14, 15).

To date, only a handful of studies have probed the mechanistic basis surrounding the beneficial neurological effects of a high fermentable fiber diet, which has the capacity to shift SCFA production to favor butyrate (16). Notably, one study found significant immune benefits in the brain of mice fed a high fermentable (soluble) fiber diet and found that they recovered faster from LPS-induced sickness (17). After exposure to LPS, mice fed the soluble fiber diet showed an increase in the IL-1 antagonist, IL-1RA, and a decrease in IL-1β and TNF-α in the brain. Brain IL-4 mRNA was also increased, and as IL-4 expression is enhanced by increased histone acetylation, the authors hypothesized that the elevated butyrate from the dietary fiber fermentation may contribute to the immune response. It is still not fully understood, however, if an increase in butyrate-producing bacteria in the gut as a consequence of soluble fiber diet, could affect microglial activation, and specifically microglial activation in aging.

Short-chain fatty acid concentrations are likely less than optimal in older adults, as data indicate daily dietary fiber intake for both older men and women is roughly 40% below the recommended adequate intake (18). There is also a lower capacity to produce butyrate in the elderly gut microbiome, as suggested by fewer copies of the butyryl-CoA:acetate CoA transferase gene compared with younger adults and lower amounts of bacterial groups which are known butyrate producers (19, 20). Therefore, the aging population is an important demographic for where increased fiber supplementation is needed. Of note, inulin is a soluble dietary fiber that increases butyrate-producing bacteria (21). Inulin belongs to a group of non-digestible carbohydrates called fructans and is considered a prebiotic (22). Studies have specifically demonstrated that inulin is beneficial for age-related inflammation, as a nutritional supplement with inulin increased innate immunity and protection against infections in elderly people (23). Thus, an increase in butyrate through added soluble fiber such as inulin could counterbalance the age-related microbiota dysbiosis, and potentially lead to neurological benefits.

We first determined if direct administration of NaB had beneficial effects on microglia in aged mice with and without an immune challenge. Building on these findings, we investigated whether soluble fiber (inulin) administered to aged mice would promote changes in the microbiome that reduced peripheral inflammation concurrent to decreased neuroinflammation. We hypothesized that NaB administration and a high soluble fiber diet could diminish or even reverse the aged neuroinflammatory phenotype in mice following an immune challenge.

## Materials and Methods

### Experiment 1: Effects of Intraperitoneal Administration of Sodium Butyrate in Adult and Aged Mice on the Microglial and Hippocampal Response to LPS

#### Animals

Adult (3- to 6-month olds) and aged (22- to 25-month olds) Balb/c male mice were reared in-house and provided with *ad libitum* access to chow (Teklad 8640, Harlan Laboratories, Indianapolis, IN, USA) and water. Mice were individually housed in a temperature-controlled environment with a 12-h reversed-phase light/dark cycle (lights on 21:00 h).

Treatment groups comprised the 2 × 2 factorial arrangement of LPS and NaB and were initiated at the onset of the dark cycle. Both *Escherichia coli* LPS (serotype 0127:B8, Sigma, St. Louis, MO, USA) and NaB (Sigma, St. Louis, MO, USA) were dissolved in sterile saline before experimentation. Mice from both age groups were injected i.p. with either saline (control) or NaB at 1.2 g/kg body weight and 2 h later with saline or LPS at 0.33 mg/kg body weight. This dose of NaB was selected based on previous studies demonstrating significant beneficial effects in other rodent models (12, 24, 25). This dose of LPS was selected based on previous studies demonstrating that 0.33 mg/kg LPS produced prolonged sickness behavior in aged compared with young mice (26). Treatments were administered at 9 a.m. for all cohorts. Body weight was measured at baseline and 4 h after injections as an index of sickness response. All studies were carried out in accordance with United States National Institutes of Health guidelines and approved by the University of Illinois Institutional Animal Care and Use Committee.

#### Tissue Collection and Microglia Isolation

Animals were euthanized *via* CO_2_ asphyxiation and transcardial perfusion with sterile ice-cold saline. Brain tissue (all but the hippocampus which was frozen in dry ice) was collected and used immediately for microglia isolation using a procedure adapted from Nikademova and Watters (27). Brains were enzymatically digested using the Neural Tissue Dissociation Kit (Miltenyi Biotec, Germany) for 35 min at 37°C. Further processing was performed at 4°C. Tissue debris was removed by passing the cell suspension through a 40-µm cell strainer. After myelin removal using 30% Percoll Plus (GE Healthcare, Princeton, NJ, USA), cells in PBS supplemented with 0.5% BSA and 2 mM EDTA were incubated for 15 min with anti-Cd11b magnetic beads (Miltenyi Biotec, Germany). CD11b^+^ cells were extensively washed and separated in a magnetic field using MS columns (Miltenyi Biotec, Germany) before being directly placed in Trizol reagent (Invitrogen).

#### RNA Isolation and Real-Time RT-PCR

Total RNA from microglia and hippocampus was isolated using the Tri Reagent protocol (Invitrogen). Synthesis of cDNA was carried out using a high-capacity RT kit (Applied Biosystems, Grand Island, NY, USA) according to the manufacturer’s instructions. Real-time RT-PCR was performed on an ABI PRISM 7900HT-sequence detection system (Perkin Elmer, Forest City, CA, USA). All genes were analyzed using PrimeTime real-time RT-PCR Assays (Integrated DNA Technologies, Coralville, IA, USA) and were compared with the housekeeping control gene GAPDH (Mm99999915_g1) using the $2^{-\Delta \Delta C_{t}}$ calculation method as previously described (28). Recent papers have established that in response to LPS, GAPDH has stable expression levels compared to other housekeeping genes (29). Based on this and on numerous other papers that have reliably used GAPDH (30–32), we chose to use GAPDH as our housekeeping gene. Data are expressed as fold change versus saline control adult mice.

#### Fluidigm

Total RNA from microglia was isolated using the Tri Reagent protocol (Invitrogen) and synthesis of cDNA was carried out using a high-capacity RT kit (Applied Biosystems) as previously described for real-time RT-PCR. Fluidigm reactions were performed by the UIUC Functional Genomics Unit of the W.M. Keck Center using a 96 × 96 chip and included two technical replicates for each combination of sample and assay. Data were acquired using the Fluidigm Real-Time PCR Analysis software 3.0.2 (Fluidigm, San Francisco, CA, USA). Data from Fluidigm runs were manually checked for reaction quality before analysis, and *C*_t_ values for each gene target (see Table 1) were normalized to *C*_t_ values for the housekeeping gene *Gapdh*.

**Table 1:** Primers used in real-time RT-PCR and Fluidigm experiments.

Gene	Assay ID
Arg1	Mm.PT.58.8651372
Casp1	Mm.PT.58.13005595
CD53	Mm.PT.58.30699738
CD68	Mm.PT.58.12034788.g
Cx3cr1	Mm.PT.58.17555544
Dnmt1	Mm.PT.58.30881142
Dnmt3a	Mm.PT.58.13545327
Dnmt3b	Mm.PT.58.31955137
Dnmt3l	Mm.PT.58.41749889
Ffar2	Mm.PT.58.32870135
Gadd45b	Mm.PT.58.10699383.g
Gapdh	Mm.PT.39a.1
Gfap	Mm.PT.58.31297710
Gpr34	Mm.PT.58.46001700
Hdac1	Mm.PT.58.43356830.g
Hdac2	Mm.PT.58.12358619
Hdac3	Mm.PT.58.11480126
Hdac4	Mm.PT.58.17651425
Hdac5	Mm.PT.58.11472897
Hdac6	Mm.PT.58.16685964
Il-1β	Mm.PT.58.41616450
Il-1rn	Mm.PT.58.43781580
Il-10	Mm.PT.58.23604055
Il-17	Mm.PT.58.6531092
Il-23	Mm.PT.58.10594618.g
Il-4	Mm.PT.58.32703659
Il-6	Mm.PT.58.13354106
Mct1	Mm.PT.58.7080950
Mecp2	Mm.PT.58.13934895.g
Muc2	Mm.PT.58.29496069.g
Nlrp3	Mm.PT.58.13974318
Ocln	Mm.PT.58.30118962
P2ry12	Mm.PT.58.43542033
P2ry13	Mm.PT.58.42597879.g
Pycard	Mm.PT.56a.42872867
Siglech	Mm.PT.58.45915252
Socs1	Mm.PT.58.11527306.g
Socs3	Mm.PT.58.7804681
Stat3	Mm.PT.58.11877007
Tet1	Mm.PT.58.43326803
Tet2	Mm.PT.58.30089849
Tet3	Mm.PT.58.11954119
Tff3	Mm.PT.58.43353357
Tgfbr1	Mm.PT.58.10230349
Tjp1	Mm.PT.58.29459730
Tjp2	Mm.PT.58.16834535
Tlr2	Mm.PT.58.45820113
Tlr4	Mm.PT.58.41643680
Tlr7	Mm.PT.58.10526075
Tlr8	Mm.PT.58.16021150
Tmem119	Mm.PT.58.6766267
Trem2	Mm.PT.58.7992121
Tnf	Mm.PT.58.12575861

### Experiment 2: Effects of Low and High Soluble Fiber Diets on Central and Peripheral Inflammation in Adult and Aged Mice

#### Animals and Diet

Adult (3- to 6-month olds) and aged (22- to 25-month olds) male Balb/c mice were housed as described in Experiment 1, but were fed either AIN-93M diet with 5% cellulose (product # D10012M, Research Diets, Inc., New Brunswick, NJ, USA) or one of two AIN-93M modified diets that contained either 1% cellulose (product # D16060605, Research Diets, Inc., New Brunswick, NJ, USA) or 1% cellulose and 5% inulin (product # D16060606, Research Diets, Inc., New Brunswick, NJ, USA) for 4 weeks. Composition of these diets can be seen in Table 2, modified from Research Diets, Inc. Fecal samples were taken from adult and aged mice (on chow) prior to the start of the study to assess the microbiome at baseline. Mice from both age groups were injected i.p. with either saline (control) or LPS at 0.33 mg/kg body weight. Treatments were administered at 9 a.m. for all cohorts. Body weight was measured at baseline and 4 h after injections as an index of sickness response. All studies were carried out in accordance with United States National Institutes of Health guidelines and approved by the University of Illinois Institutional Animal Care and Use Committee.

**Table 2:** AIN-93M mature rodent diet and modified AIN-93M diets with 10 g cellulose or 10 g cellulose + 50 g inulin per 3,850 kcal.

Product #	D10012M		D16060605		D16060606	
	*AIN-93M*		*10 g cellulose/3,850 kcal*		*10 g cellulose + 50 g inulin/3,850 kcal*	
	g %	*kcal %*	g %	*kcal %*	g %	*kcal %*
Protein	14	*15*	15	*15*	14	*15*
Carbohydrate	73	*76*	76	*76*	72	*74*
Fat	4	*9*	4	*9*	4	*9*
Total		*100*		*100*		*98*
kcal/g	3.85		4.01		3.88	

**Ingredient**	**g**	***kcal***	**g**	***kcal***	**g**	***kcal***

Casein	140	*560*	140	*560*	140	*560*
l-Cystine	1.8	*7*	1.8	*7*	1.8	*7*
Corn starch	495.692	*1,983*	495.692	*1,983*	476.942	*1,908*
Maltodextrin 10	125	*500*	125	*500*	125	*500*
Sucrose	100	*400*	100	*400*	100	*400*
Cellulose, BW200	50	*0*	10	*0*	10	*0*
Inulin	0	*0*	0	*0*	50	75
Soybean oil	40	*360*	40	*360*	40	*360*
t-Butylhydroquinone	0.008	*0*	0.008	*0*	0.008	*0*
Mineral mix S10022M	35	*0*	35	*0*	35	*0*
Vitamin mix V10037	10	*40*	10	*40*	10	*40*
Choline bitartrate	2.5	*0*	2.5	*0*	2.5	*0*
FD&C yellow dye #5	0	*0*	0	*0*	0	*0*
FD&C red dye #40	0	*0*	0.05	*0*	0.025	*0*
FD&C blue dye #1	0	*0*	0	*0*	0.025	*0*

**Total**	**1,000**	**3,850**	**960.05**	**3,850**	**991.3**	**3,850**

Cellulose (g/kg diet)	50.0		10.4		10.1	
Inulin (g/kg diet)	0.0		0.0		50.4	

#### Tissue Collection and Microglia Isolation

Four hours after LPS injections, animals were euthanized *via* CO_2_ asphyxiation, blood was taken, and animals were perfused with sterile ice-cold saline. Samples were collected from colon, liver, mesenteric lymph nodes, and hippocampus and were frozen on dry ice. Cecal content and feces were also taken for analysis of SFCA concentrations and microbiota compositions, respectively. Distal colon was taken for histological analysis. Brain tissue (all but the hippocampus) was collected and used immediately for microglia isolation as described in Experiment 1.

#### Bacterial DNA Isolation and 16S rRNA Sequencing

Fecal bacterial DNA was extracted using the PowerLyzer PowerSoil DNA Isolation Kit (MOBIO Laboratories, Inc.) and quality was assessed *via* gel electrophoresis. The DNA library was constructed using a Fluidigm Access Array system in the Functional Genomics Unit of the Roy J. Carver Biotechnology Center at the University of Illinois at Urbana (UIUC). After library construction, a 250-bp region of the 16S rRNA gene (V4) were amplified using a PCR amplification method modified from Muturi et al. (33). Briefly, DNA samples were diluted to 2 ng/µL and amplified with Roche High Fidelity Fast Start Kit and 20× Access Array loading reagent prior to PCR. Samples were amplified using the following Access Array cycling program 50°C for 2 min (1 cycle), 70°C for 20 min (1 cycle), 95°C for 10 min (1 cycle), followed by 10 cycles at 95°C for 15 s, 60°C for 30 s, and 72°C for 1 min, 2 cycles at 95°C for 15 s, 80°C for 30 s, 60°C for 30 s, and 72°C for 1 min, 8 cycles at 95°C for 15 s, 60°C for 30 s, and 72° for 1 min, 2 cycles at 95°C for 15 s, 80°C for 30 s, 60°C for 30 s, and 72°C for 1 min, 8 cycles at 95°C for 15 s, 60°C for 30 s, and 72°C for 1 min, and 5 cycles at 95°C for 15 s. After PCR, all samples were run on a Fragment Analyzer (Advanced Analytics, Ames, IA, USA). Samples were then pooled in equal amounts according to PCR product concentration. The pooled products were then size selected on a 2% agarose E-gel (Life Technologies) and extracted from the isolated gel slice with Qiagen gel extraction kit (Qiagen). Cleaned size selected products were run on an Agilent Bioanalyzer to confirm appropriate profile and determination of average size. The final library pool was spiked with 10% non-indexed PhiX control library (Illumina^®^) and sequenced using Illumina^®^ MiSeq^®^ V3 Bulk system. The libraries were sequenced from both ends of the molecules to a total read length of 250 nt from each end. High-quality (>25) sequence data (FASTQ) from the forward read were analyzed with QIIME 2.0 (34). Quality control consisted of depleting or removing barcodes, primers, and short sequences (<187 bp), sequences with ambiguous base calls, and sequences with homopolymer runs exceeding 6 bp. After removal of singletons, OTUs were classified using closed reference picking against Greengenes database at 97% similarity. β-diversity (weighted and unweighted UniFrac distances) were computed at an even sampling depth of 10,048 sequences per sample based on α-diversity (Chao1) rarefaction plots (Figure S1 in Supplementary Material).

#### SCFA Analysis

Short-chain fatty acids were analyzed by dry matter content as described previously by Panasevich et al. (35), courtesy of the Metabolomics Center at the University of Illinois at Urbana-Champaign. To calculate dry matter percentage, a portion (~0.08–0.15 g) of wet cecal contents were weighed and then heated in oven for 24 h to dry samples. Resulting dry matter percentage was calculated as [weight of dry sample/weight (g) of wet sample (g) × 100]. The rest of the cecal contents (~0.05–0.15 g) were acidified immediately after collection in 6.25% meta-phosphoric acid solution and stored at −20°C until analysis. SCFA concentrations (wet) were determined by gas chromatography by using a gas chromatograph (Hewlett-Packard 5890A Series II) and a glass column (180 cm × 4 mm i.d.), packed with 10% SP-1200/1% H3PO4 on 80/100 + mesh Chromosorb WAW (Supelco, Inc.). Nitrogen was the carrier gas with a flow rate of 75 mL/min. Oven, detector, and injector temperatures were 125°, 175°, and 180°C, respectively. Acetic, *n*-butyric, and propionic acid solutions (Sigma-Aldrich) were used as standards.

#### RNA Isolation and Real-Time RT-PCR

Total RNA from microglia, hippocampus, colon, liver, and mesenteric lymph nodes was isolated and synthesis of cDNA was carried out as described in Experiment 1. Real-time RT-PCR was performed also as described in Experiment 1 with the exception that data are expressed as fold change versus control adult mice on AIN-93M diet.

#### Fluidigm

As described in Experiment 1.

#### DNA Isolation and Pyrosequencing

Total DNA from microglia and hippocampus tissue was isolated using the Tri Reagent protocol (Invitrogen). DNA methylation for 2 5′—C—phosphate—G—3′ (CpG) sites within the proximal promoter region of IL-1β was assessed *via* bisulfite pyrosequencing on bisulfite modified DNA (Zymo Research, Irvine, CA, USA). The mouse IL-1β methylation assay (ID ADS3713-RS1) was purchased from EpigenDx (Hopkinton, MA, USA) and has been previously used to assess methylation status in microglia (36). PCRs were run in duplicate and contained 20 ng of bisulfite converted DNA as starting template. Product specificity was determined by gel electrophoresis. The primer was also tested using bisulfite converted DNA from high and low methylation controls (EpigenDx, Hopkinton, MA, USA). Qiagen’s PyroMark Q24 Advanced Pyrosequencer was used to detect DNA methylation levels following manufacturer’s protocols and default settings (Qiagen, Valencia, CA, USA), similar to a previous study (37).

#### Distal Colon Histology

Distal colon was fixed in Methacarn (60% methanol, 30% chloroform, and 10% acetic acid) for 72 h and then placed in 100% ethanol until tissue embedding. Distal colon tissue was paraffin embedded, and 5-µm sections mounted on glass slides were stained with hematoxylin and eosin (H&E) for assessment of intestinal morphology, courtesy of the Histology Laboratory in the College of Veterinary Medicine at University of Illinois at Urbana-Champaign. Histology was scored by a trained, blinded observer on a modified version of a published scoring (38) system that considers four criteria: inflammatory cell infiltration (0–4), goblet cell mucus depletion (0–4), destruction of architecture (0–4), and crypt abscesses (0–4). Overall histology score was quantified using an aggregate of these scores.

#### LPS Binding Protein (LBP) Assay

LPS binding protein was quantified in serum samples using the Mouse LBP enzyme-linked immunosorbent assay Kit (catalog #: HK205-02, Hycult Biotech Inc., Plymouth Meeting, PA, USA). The assay was run according to the manufacturer’s instructions.

#### Statistical Analyses

Body weight, gene expression (including Fluidigm), SCFA analysis, pyrosequencing, colon histology scoring, and LBP data were analyzed using Statistical Analysis System (SAS, Cary, NC, USA) and were subjected to two-way analysis of variance (ANOVA). Where ANOVA revealed a significant interaction (*p* < 0.05), Fisher’s LSD test was used for *post hoc* comparisons. Microbiome community compositions (β-diversity; weighted Unifrac) were compared across ages and diets with permutational analysis of variance analysis (PERMANOVA). Next, to unbiasedly identify key features of the gut microbiota that are affected by age and fiber diets, Random Forest (RF) alongside Boruta feature selection was performed. Boruta selection uses RF to iteratively compare importance of independent variables with that of pseudo-random (shadow) attributes (39). Variables “confirmed” by Boruta feature selection had significantly higher RF importance scores than “shadow” attributes and could therefore delineate between diet and age. Analysis was performed on R RF package with 1,000 trees and all other default values. All data are expressed as mean ± SEM.

## Results

### Sodium Butyrate Downregulates Il-1β Gene Expression in Response to LPS in Adult and Aged Microglia and Hippocampus

To test the effects of NaB on LPS-induced neuroinflammation, adult and aged mice were either pretreated i.p. with saline (SAL) or NaB, and then given an i.p. injection of either SAL or LPS 2 h later. For body weight, LPS-treated adult and aged mice lost more weight than saline-treated controls as expected (main effect of LPS, *p* = 0.0052 and *p* < 0.0001), but there was also an interaction (NaB × LPS, *p* = 0.0391) with the aged mice in that NaB pretreated mice with LPS did not lose as much weight as the SAL pretreated mice with LPS (data not shown).

**Figure 1:**
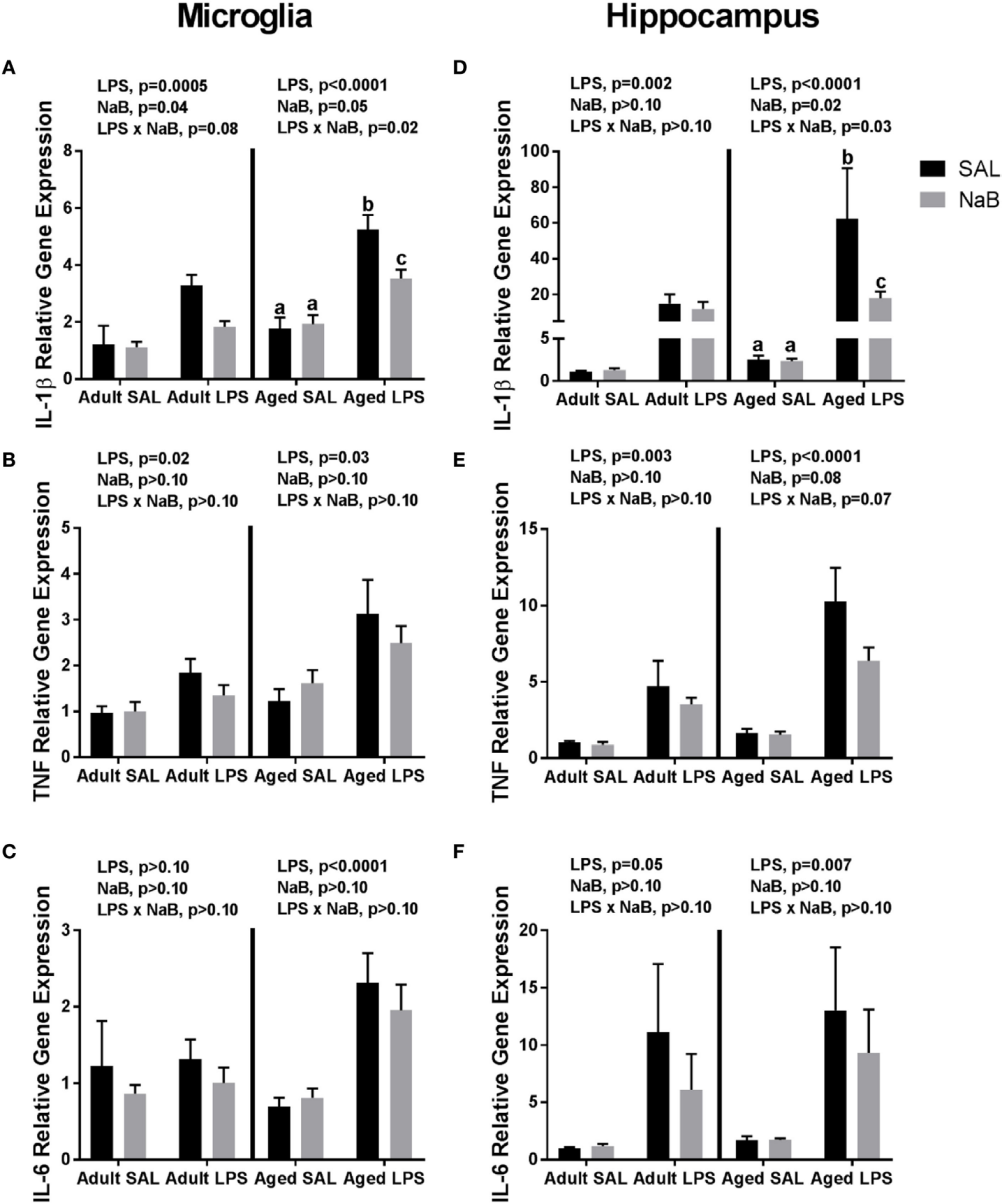
Microglial and hippocampal gene expression 4 h after SAL/lipopolysaccharide (LPS) i.p. injections in adult and aged mice pretreated with i.p. SAL/NaB. Data are presented as mean ± SEM (*n* = 7–10) for microglial **(A)** *Il-1β*, **(B)** *Tnf*, **(C)** *Il-6* and hippocampal **(D)** *Il-1β*, **(E)** *Tnf*, and **(F)** *Il-6* gene expression. Treatment means with different letters are significantly different at *p* < 0.05.

Fluidigm gene expression analysis on microglia was performed to gain a more comprehensive assessment of microglial gene regulation in response to NaB (Tables S1 and S2 in Supplementary Material). However, there were very few changes associated with NaB in the majority of genes analyzed, so we chose to focus on pro-inflammatory cytokine gene expression (Figures 1A–C). In adult microglia, immune activation by LPS increased *Il-1β*, and butyrate attenuated *Il-1β* expression [main effects of LPS (*p* = 0.0005), NaB (*p* = 0.0447), and a trending interaction (*p* = 0.0774)]. An interesting finding was that for aged microglia, there were more pronounced changes in *Il-1β* in that NaB decreased LPS-induced *Il-1β* expression [main effects of LPS (*p* < 0.0001), NaB (*p* = 0.0497), and an interaction (*p* = 0.0178)]. LPS increased microglial *Tnf* in both adult and aged mice (main effects of LPS, *p* = 0.02 and *p* = 0.003), and LPS increased microglial *Il-6* in only aged mice (main effect of LPS, *p* < 0.0001).

Similar changes were also observed in the hippocampus (Figures 1D–F), a brain region that is particularly sensitive to the effects of neuroinflammation (40). In the adult hippocampus, LPS increased *Il-1β* (main effect of LPS, *p* = 0.0018). But in the aged hippocampus, LPS increased *Il-1β* and NaB attenuated LPS-induced *Il-1* [main effects of LPS (*p* < 0.0001), NaB (*p* = 0.0246), and an interaction (*p* = 0.0257)]. For hippocampal *Tnf* and *Il-6*, LPS increased their expression in both adult and aged mice (main effects of LPS, *p* = 0.0031 and *p* < 0.0001, and *p* = 0.0475 and *p* = 0.0069), although in aged mice there were trends for a NaB main effect and interaction for *Tnf* (*p* = 0.0804 and *p* = 0.0651).

### Significant Shifts in the Microbiome Associated With Age

Microbiome analysis was performed to identify differences between adult and aged mice at baseline. β-diversity analysis revealed that gut microbiota composition was different between adult (3–6 months) and aged (22–25 months) mice fed a normal chow diet (PERMANOVA *p* < 0.05). RF analysis with Boruta feature selection identified lower levels of *Mucuspirillum* spp. and *Odoribacter* spp., and higher levels of in *Ruminococcus* spp., *Coprococcus* spp., and *Rikenellaceae* in aged versus adult mice.

### High Soluble Fiber Diet Alters Microbiota and Increases Production of SCFAs

Adult and aged mice were fed either a low fiber (1% cellulose) or high fiber (1% cellulose + 5% inulin) diet for 4 weeks, and microbiome analysis from fecal samples was performed to identify changes from baseline and confirm previous effects of high fiber diet on the microbiome. Mice were injected i.p. with SAL or LPS, but we chose to focus on SAL animals, so all results presented here are in SAL animals unless otherwise stated. For body weight, there were no differences in body weight for age or diet (*p* > 0.05, data not shown).

**Figure 2:**
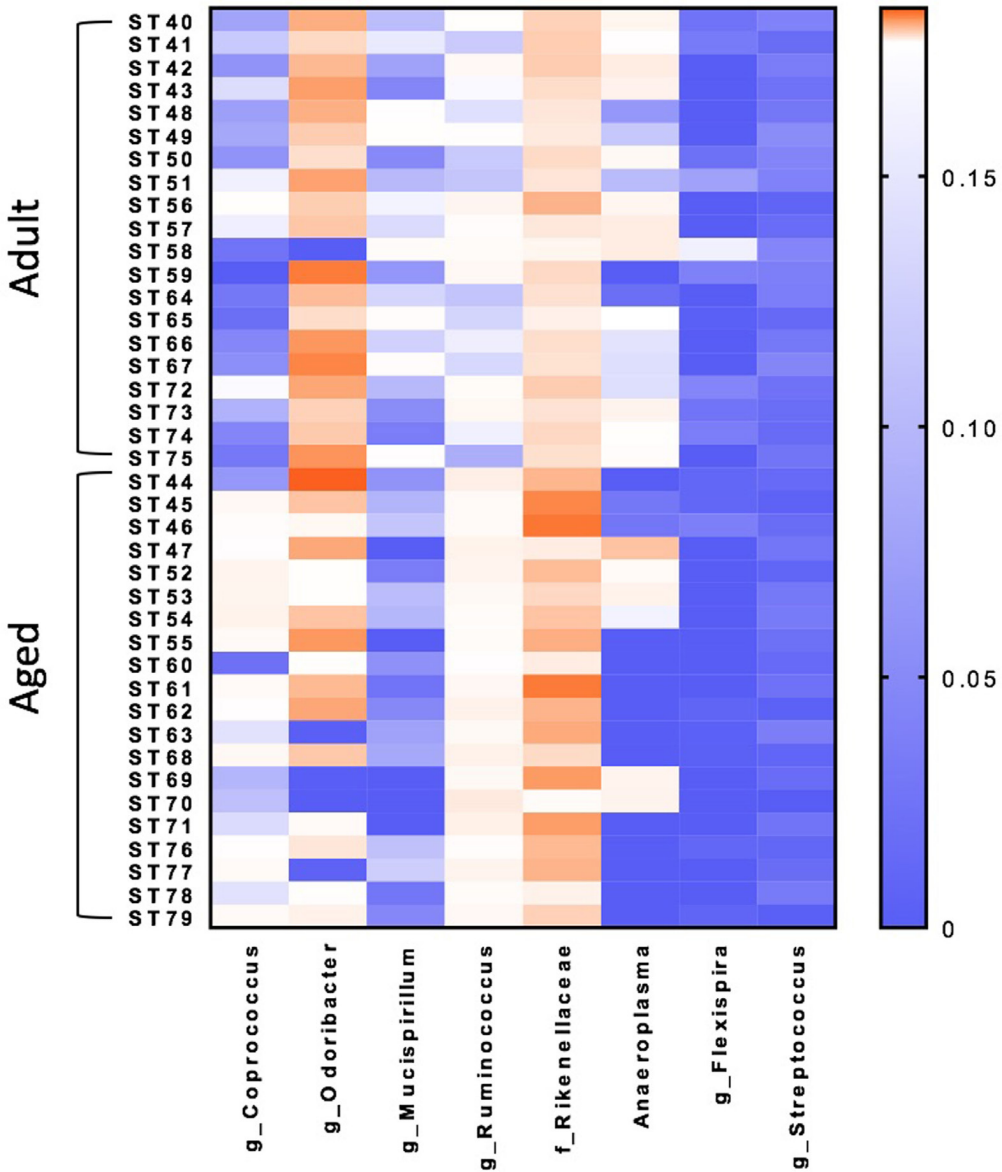
Random forest analysis with Boruta feature identifies eight bacterial genera within the fecal microbiome that discriminate between adult and aged mice fed a normal chow diet. *N* = 20 per group. Scale represents relative abundance.

**Figure 3:**
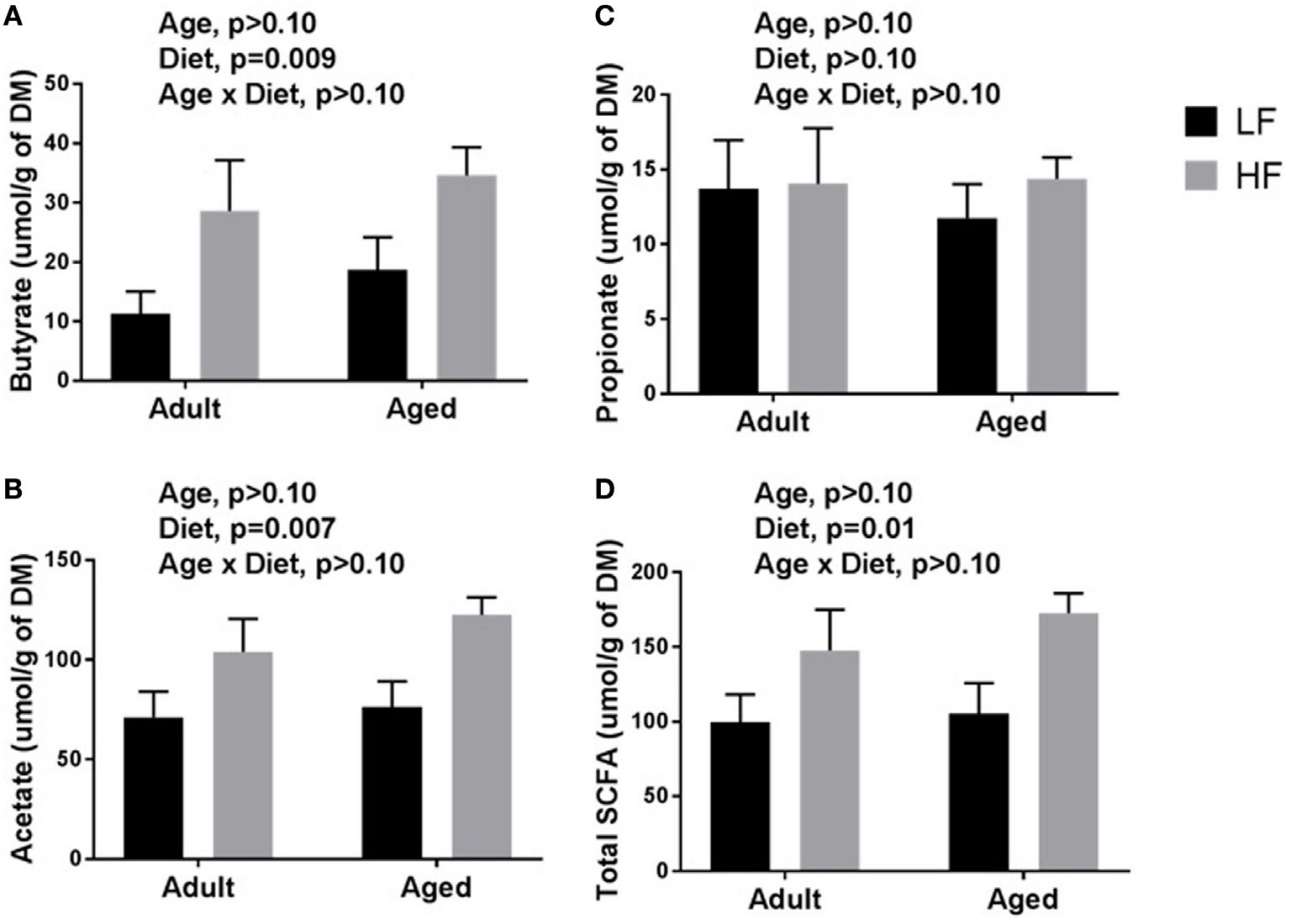
Cecal content concentrations of **(A)** butyrate, **(B)** acetate, **(C)** propionate, and **(D)** total short-chain fatty acids (SCFAs) in adult and aged mice fed a LF (1% cellulose) or HF (1% cellulose and 5% inulin) diet. Data are presented as mean ± SEM (*n* = 6–8).

β-diversity analysis revealed a robust effect of just 4 weeks of soluble fiber feeding on gut microbiota composition (PERMANOVA *p* < 0.05; Figure S2 in Supplementary Material). Of note, inulin feeding led to lower abundances of *Ruminococcus* spp. and f. Rikenellaceae, two taxa that were more highly represented in aged mice fed a chow diet (Figure 2). Cecal contents were analyzed for SCFA concentrations to assess the magnitude and profile of fermentation end products (Figure 3). Inulin feeding led to an increase in cecal acetate (*p* = 0.0066), butyrate (*p* = 0.009), and total SCFAs (*p* = 0.0107), irrespective of age. Propionate concentrations were not responsive to diet nor affected by age (*p* > 0.05; Figure 3C).

### Soluble Fiber Diet Reduces Colonic Inflammation but Does Not Rescue Signs of Systemic Endotoxemia in Aged Mice

**Figure 4:**
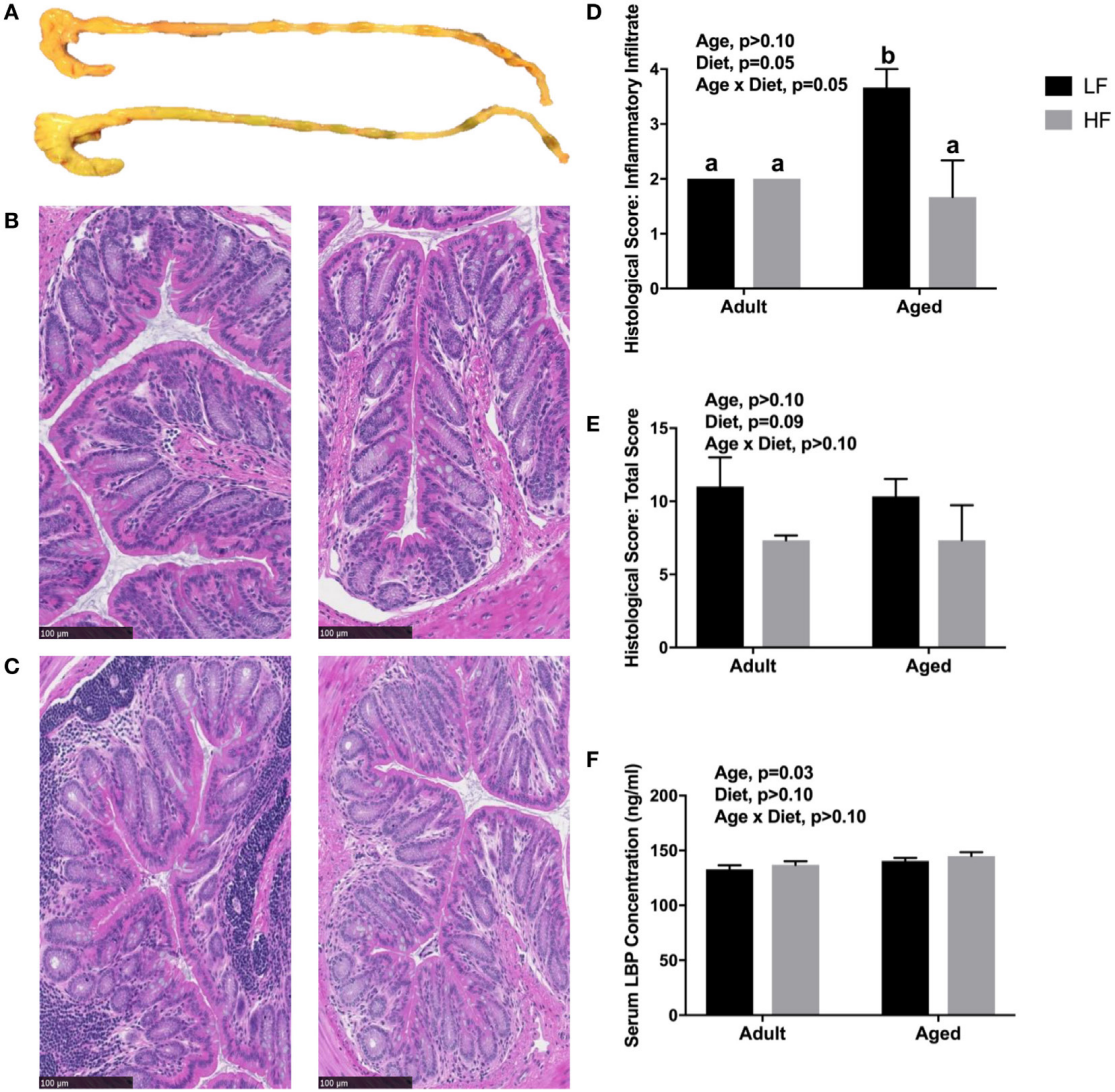
**(A)** Representative images of cecum and colon in mice fed either a low (top) or high (bottom) fiber diet. Representative sections of the distal colon of 4-month-old **(B)** and 24-month-old **(C)** mice fed either a low (left) or a high (right) diet. Sections were stained with hematoxylin and eosin. Scale bar = 100 µM. Blinded histological scoring of distal colons: **(D)** inflammatory cell infiltration (score 0–4) and **(E)** total histology score (0–16) [includes aggregate scores of inflammatory cell infiltration (0–4), goblet cell mucus depletion (0–4; data not shown), destruction of architecture (0–4; data not shown), and crypt abscesses (0–4; data not shown)]. Data are presented as mean ± SEM (*n* = 3). Bars with no SEM indicate identical scoring for all mice in the group. Treatment means with different letters are significantly different at *p* < 0.05. **(F)** Serum LPS binding protein (LBP) measured in adult and aged mice fed low or high fiber diets. Data are presented as mean ± SEM (*n* = 7–9).

Since we observed changes in the microbiome and SCFA analysis with adult and aged mice fed different fiber diets, we hypothesized that high fiber would reduce signs of inflammation within the periphery. Representative images of the cecum and colon from mice fed low fiber and high fiber diets revealed increased cecal size with the high fiber diet, indicative of increased fermentation (Figure 4A). Distal colon samples were stained with H&E to examine morphological changes due to age and fiber diet. Representative pictures (Figures 4B,C) indicated severe inflammation associated with immune infiltration in aged animals on a low fiber diet. Scoring of histology sections for inflammatory infiltrate reveal that there was a main effect of diet (*p* = 0.0499) and an interaction (*p* = 0.0499) in that aged mice on the high fiber diet had decreased inflammatory infiltrate, with scoring not significantly different from either low fiber or high fiber adult groups (Figure 4D). With regards to total histological scoring, there was a trending main effect for diet (*p* = 0.088) in that diet decreased total score (Figure 4E). Lastly, we assessed LBP in serum of adult and aged animals fed low and high fiber diets as an indirect measure of gut permeability (41). LBP was increased by age (*p* = 0.0298) which matches previous findings from our lab (unpublished results), but no significant effect of diet (Figure 4F).

### Tight Junctions Regulating Colonic Barrier Function Are Higher in Aged Mice Fed a High Fiber Diet

**Figure 5:**
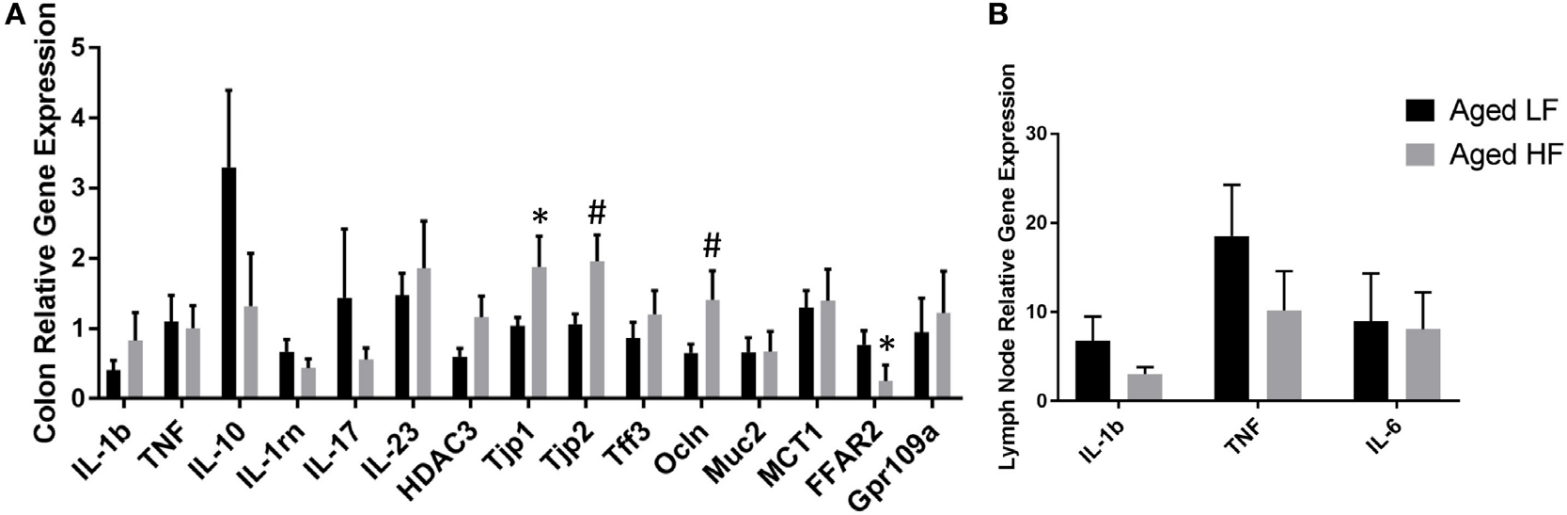
Gene expression of **(A)** colon and **(B)** lymph nodes from aged mice fed either a low or high fiber diet. Data are presented as mean ± SEM (*n* = 5–7). * indicates significance at *p* < 0.05 and ^#^ indicates significance at *p* < 0.1.

Butyrate and other SCFAs exert their effects through HDAC inhibition, but are also used as an energy source and can bind G protein-coupled receptors (GPCRs) that are involved in the resolution of inflammation in the gut (42). Therefore, gene expression for the pro-inflammatory cytokines *Il-1β* and *Tnf*, other inflammatory regulators including *Il-10* and *Il-1rn, Il-17*, and *Il-23*, the HDAC *Hdac3*, tight junction proteins/intestinal markers *Tjp1, Tjp2, Tff3, Ocln, Mct1*, and *Muc2*, as well as the FFARs *Ffar2* and *Gpr109a* were measured in colon (Figure 5A). There were no significant differences in any of the genes analyzed in adult mice (data not shown), so we chose to focus on aged animals. Surprisingly, there were no effects of diet on pro-inflammatory gene expression, but there was an effect of diet for *Tjp2* (*p* = 0.0396) and *Ffar2* (*p* = 0.0319). There were also trending effects of diet for *Tjp1* (0.0738) and *Ocln* (*p* = 0.0857). Gene expression for the pro-inflammatory cytokines *Il-1β, Tnf*, and *Il-6* were assessed in lymph nodes and liver, but there were no differences between low fiber and high fiber groups in adult (data no shown) or aged mice (Figure 5B). However, numerically there was lower expression of the pro-inflammatory cytokines in lymph nodes of aged animals on the high fiber diet.

### Fiber Alters Expression of Inflammatory, Sensome, and Epigenetic Regulator Genes in Adult and Aged Microglia

**Figure 6:**
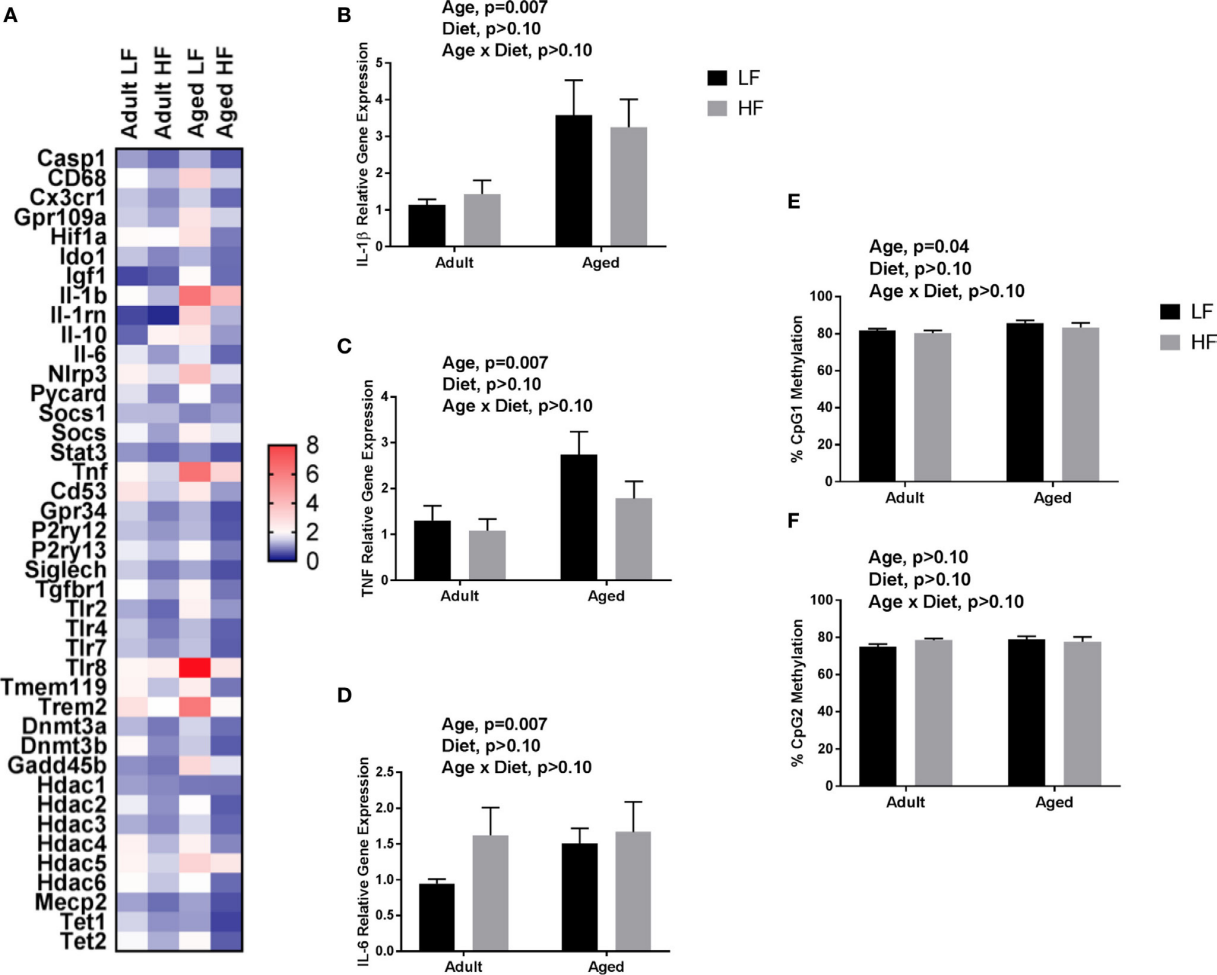
**(A)** Heat map visualization of relative expression of genes in microglia analyzed by Fluidigm. Microglia were collected from adult and aged mice fed a LF (1% cellulose) or HF (1% cellulose and 5% inulin) diet. Hippocampal **(B)** *Il-1β*, **(C)** *Tnf*, **(D)** *Il-6* gene expression and DNA methylation of the *Il-1β* promoter at **(E)** CpG1 and **(F)** CpG2. Data are presented as mean ± SEM (*n* = 6–8).

We next wanted to determine whether beneficial changes in the periphery through high fiber diet could affect microglial gene regulation (Figure 6A). For inflammatory and regulators of inflammatory genes, Fluidigm analysis indicated that there were a number of significant main effects of diet in that high fiber decreased gene expression (*Il-1β, Il-1rn, Il-6, Nlrp3, Tlr4*, and *Tnf*) (Table 3). For sensome genes, there were main effects of diet for all genes analyzed (Table 3), as well as an interaction with *Trem2* in which aged mice on the low fiber diet had the highest expression but decreased to levels similar to the adult groups with the high fiber diet. For epigenetic regulator genes, there were also main effects of almost all genes analyzed, in that high fiber decreased expression (Table 3). Of note, some epigenetic regulator genes were affected by age similar to what has been demonstrated previously (*Hdac1* and *Gadd45b*) (3).

**Table 3:** Expression of (A) inflammatory, (B) sensome, and (C) epigenetic regulator genes in microglia collected from adult and aged mice fed low or high fiber diets.

					*p*-Values		
Gene	Adult LF	Adult HF	Aged LF	Aged HF	Age	Diet	Age × DIET
**(A) Inflammatory and regulators of inflammatory genes**							
Casp1	1.10 ± 0.15	0.68 ± 0.12	1.28 ± 0.15	0.59 ± 0.09	0.734	<0.0001*	0.315
Cd68	1.80 ± 0.32	1.27 ± 0.22	3.24 ± 0.46	1.42 ± 0.32	0.025*	0.0012*	0.067
Cx3cr1	1.38 ± 0.22	0.96 ± 0.21	1.46 ± 0.23	0.72 ± 0.17	0.695	0.0079*	0.447
HIF-1α	1.96 ± 0.35	1.89 ± 0.59	2.74 ± 0.33	0.85 ± 0.15	0.753	0.019*	0.028*
Ido1	1.37 ± 0.21	0.94 ± 0.27	1.26 ± 0.17	0.76 ± 0.16	0.491	0.0279*	0.872
Igf1	0.49 ± 0.09	0.68 ± 0.22	1.95 ± 0.30	0.74 ± 0.15	0.0013*	0.0276*	0.0027*
Il-1β	1.91 ± 0.30	1.30 ± 0.29	6.34 ± 0.68	4.03 ± 0.84	<0.0001*	0.012*	0.134
Il-1rn	0.48 ± 0.06	0.25 ± 0.07	3.35 ± 3.51	1.26 ± 0.19	<0.0001*	0.0002*	0.0023*
Il-10	0.70 ± 0.18	2.22 ± 0.64	2.57 ± 0.48	1.07 ± 0.32	0.512	0.989	0.0094*
Il-6	1.62 ± 0.17	1.08 ± 0.23	1.63 ± 0.17	0.70 ± 0.14	0.327	0.0002*	0.300
Niarc1	1.44 ± 0.15	1.13 ± 0.08	2.65 ± 0.30	1.46 ± 0.20	0.0002*	0.0004*	0.0293*
Nlrp3	2.24 ± 0.27	1.55 ± 0.39	3.87 ± 0.56	1.57 ± 0.40	0.060	0.001*	0.066
Pycard	1.58 ± 0.25	0.93 ± 0.19	1.87 ± 0.25	0.91 ± 0.19	0.539	0.0007*	0.506
Socs1	1.30 ± 0.08	1.29 ± 0.05	0.93 ± 0.11	1.14 ± 0.06	0.0022*	0.246	0.187
Socs3	1.71 ± 0.30	1.11 ± 0.27	2.27 ± 0.44	1.60 ± 0.51	0.181	0.105	0.918
Stat3	1.03 ± 0.16	0.72 ± 0.13	1.04 ± 0.13	0.58 ± 0.10	0.617	0.0051*	0.577
Tlr2	1.21 ± 0.19	0.73 ± 0.14	2.24 ± 0.33	1.04 ± 0.22	0.0048*	0.0006*	0.125
Tlr4	1.40 ± 0.17	0.85 ± 0.18	1.31 ± 0.17	0.67 ± 0.13	0.419	0.0006*	0.762
Tlr7	1.35 ± 0.14	1.02 ± 0.24	1.35 ± 0.17	0.65 ± 0.11	0.302	0.0048*	0.293
Tlr8	2.10 ± 0.46	2.30 ± 0.59	8.72 ± 1.55	2.60 ± 0.52	0.089	0.113	0.013*
Tnf	2.09 ± 0.29	1.46 ± 0.47	6.42 ± 0.93	3.17 ± 1.03	0.0001*	0.011*	0.081

**(B) Sensome genes**							
Cd53	2.67 ± 0.59	1.40 ± 0.28	2.53 ± 0.47	1.08 ± 0.21	0.602	0.003*	0.851
Gpr34	1.46 ± 0.16	0.87 ± 0.22	1.26 ± 0.15	0.54 ± 0.11	0.119	0.0003*	0.698
P2ry12	1.05 ± 0.22	1.05 ± 0.22	1.29 ± 0.17	0.60 ± 0.11	0.134	0.005*	0.267
P2ry13	1.66 ± 0.28	1.26 ± 0.30	1.95 ± 0.31	0.87 ± 0.21	0.876	0.011*	0.231
Siglech	1.43 ± 0.16	0.81 ± 0.18	1.18 ± 0.14	0.54 ± 0.11	0.089	<0.0001*	0.933
Tgfbr1	1.84 ± 0.32	1.14 ± 0.22	2.10 ± 0.28	0.81 ± 0.19	0.891	0.0003*	0.262
Tmem119	2.15 ± 0.38	1.36 ± 0.38	2.36 ± 0.34	0.82 ± 0.25	0.636	0.0014*	0.283
Trem2	2.80 ± 0.61	1.82 ± 0.33	6.23 ± 1.04	1.99 ± 0.53	0.011*	0.0004*	0.021*

**(C) Epigenetic regulator genes**							
Dnmt3a	1.28 ± 0.19	0.83 ± 0.18	1.49 ± 0.18	0.77 ± 0.16	0.681	0.0019*	0.456
Dnmt3b	2.01 ± 0.31	0.96 ± 0.20	1.41 ± 0.21	0.63 ± 0.12	0.046*	0.0002*	0.566
Gadd45b	1.01 ± 0.12	0.81 ± 0.22	3.04 ± 0.35	1.60 ± 0.40	<0.0001*	0.006*	0.033*
Hdac1	1.11 ± 0.02	0.95 ± 0.07	0.83 ± 0.04	0.82 ± 0.03	<0.0001*	0.068	0.106
Hdac2	1.67 ± 0.30	1.00 ± 0.22	1.87 ± 0.23	0.63 ± 0.14	0.704	0.0001*	0.224
Hdac3	1.22 ± 0.17	0.94 ± 0.18	1.50 ± 0.20	0.71 ± 0.12	0.855	0.0029*	0.145
Hdac4	2.20 ± 0.47	1.27 ± 0.33	2.25 ± 0.38	0.93 ± 0.73	0.699	0.0037*	0.595
Hdac5	2.13 ± 0.38	1.48 ± 0.41	3.18 ± 0.65	2.60 ± 1.04	0.100	0.350	0.957
Hdac6	1.91 ± 0.27	1.38 ± 0.39	1.86 ± 0.29	0.74 ± 0.17	0.256	0.0085*	0.331
Mecp2	1.13 ± 0.17	0.77 ± 0.16	1.13 ± 0.17	0.54 ± 0.10	0.429	0.0019*	0.457
Tet1	1.49 ± 0.17	1.01 ± 0.20	1.09 ± 0.15	0.44 ± 0.09	0.004*	0.001*	0.609
Tet2	1.75 ± 0.27	1.22 ± 0.23	1.97 ± 0.24	0.64 ± 0.10	0.422	0.0001*	0.080
Tet3	3.54 ± 0.70	1.90 ± 0.50	5.55 ± 1.09	5.55 ± 2.23	0.021*	0.488	0.489

*Data are presented as mean ± SEM (*n* = 7–10) and *p*-values for main effects of age and diet as well as age × diet interactions are also included*.

*indicates significance at p < 0.05.

We also wanted to determine if the increased peripheral butyrate as a result of a fiber diet is related to central anti-inflammatory effects. Here, we observed that microglial expression of *Il-1β, Tnf*, and *Il-6* inversely associated with cecal butyrate concentrations (data not shown). However, higher acetate and total SCFA concentrations were also inversely associated with *Il-1β, Tnf*, and *Il-6*, and propionate was only significantly correlated with *Il-1β* (data not shown).

### Fiber Does Not Alter Expression of Inflammatory Genes or *Il-1β* DNA Methylation in Adult and Aged Hippocampus

Only main effects of age were observed in the hippocampus of adult and aged mice fed low and high fiber diets, in that age increased pro-inflammatory gene expression (*Il-1β, p* = 0.0074, *Tnf, p* = 0.0145, and *Il-6, p* > 0.05) (Figures 6B–D). Recently, it has been found that SCFA can modify not only histone modifications, but also DNA methylation. Therefore, we chose to assess the effects of fiber diet on DNA methylation patterns within the brain. Due to low DNA yield, we were not able to assess *Il-1β* DNA methylation in microglia. However, in the hippocampus (Figures 6E,F), analysis of *Il-1β* DNA methylation revealed a main effect of age in *Il-1β* CpG1 (*p* = 0.0395). There were no changes in *Il-1β* CpG2.

### Differences in Central Immunoreactivity Is Minimal Between Mice Fed Low and High Fiber Diets

For Experiment 2, a separate cohort of mice was injected i.p. with LPS to characterize differences in immune stimulus associated with diet. LPS decreased body weight as expected (1–2 g on average, data not shown), differences in body weight existed with respect to age or diet (*p* > 0.05, data not shown).

Fluidigm analysis from microglia is shown in Table S3 in Supplementary Material. Interestingly, the majority of the diet main effects and interactions were seen in the sensome (*Cd53, Siglech, P2ry12, P2ry13, Trem2*, and *Tmem119*). For the hippocampus, only one main effect of age was observed in adult and aged mice fed low and high fiber diets (*Il-1β, p* = 0.0325, *Tnf, p* = > 0.05, and *Il-6, p* > 0.05) (Figure S3 in Supplementary Material). Due to low DNA yield, we were not able to assess *Il-1β* DNA methylation in microglia. However, in the hippocampus (Figure S3 in Supplementary Material), analysis of *Il-1β* DNA methylation revealed a main effect of age in *Il-1β* CpG2 (*p* = 0.001) and an interaction (*p* = 0.0064) in that aged mice had less DNA methylation than both adult groups but adult mice with the high fiber diet had increased DNA methylation compared with adult mice with the low fiber diet. There were no differences as a result of diet and age in *Il-1β* CpG1. With regards to the periphery, colon, lymph nodes, and liver gene expression were analyzed in the same way as Figure 5. The only effect of diet was *Ffar2* colon gene expression (*p* = 0.0179), in that *Ffar2* increased in aged animals with the high fiber compared with the low fiber diet (Figure S4 in Supplementary Material). There were also no differences in LBP (Figure S3 in Supplementary Material).

## Discussion

These studies tested the hypotheses that (1) peripheral administration of NaB attenuates basal inflammation and immune reactivity in aged microglia, (2) a high soluble fiber diet, through production of butyrate, attenuates peripheral and central inflammation associated with aging, and that (3) a high soluble fiber diet attenuates aging-induced microglial hyperactivity observed in response to an immune challenge.

First, we demonstrated that LPS-induced *Il-1β* gene expression was decreased in microglia and hippocampus in NaB-pretreated aged mice. There were similar trends in adult mice, but surprisingly, effects were much more pronounced in the aged. It has been found in other models that aged animals are more responsive to the effects of butyrate (24, 43), and it is suggested that divergent gene expression and behavioral patterns in adult and aged animals from HDACi treatment may be due to differential chromatin modifications within the brain (44). Furthermore, as some of these effects of butyrate occur with improvements in metabolism, it may be possible that benefits are more pronounced in the aged because butyrate is able to counteract aspects of age-related metabolic dysfunction that is not seen in adult animals. Nonetheless, our results support the hypothesis that butyrate can attenuate pro-inflammatory cytokine expression in microglia, similar to previous findings (7, 8). Because NaB exerted an anti-inflammatory effect on microglia in our aged mouse model, we wanted to determine whether an increase in butyrate through a high soluble fiber diet had the potential to alter neuroinflammation.

It has previously been observed that there are distinct microbiomes in aged versus young mice, a finding that has been reported to be related to aging-induced inflammation (45, 46). Of note, there were lower levels of *Mucuspirillum* spp. and *Odoribacter* spp., and higher levels of in *Ruminococcus* spp., *Coprococcus* spp., and *Rikenellaceae* in aged versus adult mice, even though the mice were from the same colony, maintained in the same room, and fed an identical diet. Higher levels of *Ruminococcus* spp. have been observed in elderly humans and were associated with high frailty scores (47), while higher levels of *Rikenellaceae* have been shown in aged mice compared with adult mice (48). Interestingly, high fiber diets decreased both *Ruminococcus* spp. and *Rikenellaceae*, indicative of a potential beneficial effect. Surprisingly, we saw no differences in the butyryl-CoA:acetate CoA transferase (data not shown), a gene that is thought to be a valuable marker for gastrointestinal microbiota function and increased butyrate production/butyrate-producing bacteria (20). However, SCFA analysis revealed higher acetate and butyrate production in response to a high fiber diet irrespective to any significant changes in SCFA producing genes. This discrepancy in results may be due to the fact that mice were only on low and high fiber diets for 4 weeks. A longer diet intervention may be necessary for more pronounced changes in SCFA gene abundance. Furthermore, since SCFAs are essential metabolic regulators of the microbiome, intermediary metabolites such as lactate and succinate should also be considered to play a role, and optimizing SCFA production may require other dietary manipulations (49).

Colon histology revealed significant immune infiltration within the colon lamina propria of mice fed a lower-fiber diet, while aged mice on the high fiber diet exhibited relatively lower inflammatory infiltrate. Such an effect was not observed in adult mice. This demonstrates that a low fiber diet creates an inflammatory environment that is more pronounced in aged, and that a high fiber diet has the potential to limit colonic inflammation.

A surprising finding was that although fiber led to attenuated pro-inflammatory gene expression in the brain, these findings were not directly mirrored in the colon, lymph nodes, or liver. This could be due to length of the diet, or to the heterogeneous mix of cells within these tissues. Interestingly, in a recent study with pigs, oral NaB after 4 weeks changed regional brain glucose metabolism and hippocampal neurogenesis, with little effects on the gut, demonstrating CNS-specific effects due to butyrate (50). There were, however, effects of diet in the colon for *Tjp2* and trending effects for *Tjp1* and *Ocln* in that high fiber diet increased all of the genes, indicating a potential beneficial change in gut permeability. These findings concur with previous studies indicating the ability of butyrate to upregulate tight junction proteins (51). There was also a significant decrease in *Ffar2*, which may be related to decreased neutrophil infiltration (52). Emerging evidence indicates that the microbiome regulates neutrophil homeostasis and specifically that SCFAs regulates neutrophil-mediated inflammation (53). Moreover, with the greater understanding of neutrophil populations within the brain (54), and the fact that neutrophils have been demonstrated to promote Alzheimer’s like pathology and cognitive decline (55), this cell type is likely to play an important role in cell trafficking in response to SCFAs in the brain. Further studies are warranted to determine whether an altered microbiome due to aging, inflammation, or diet could induce changes in neutrophil phenotype, and whether targeting neutrophils has an effect on the microbiome and brain.

Fiber was effective in limiting central inflammation associated with age, evidenced by lower microglial inflammatory gene expression (*Il-1β, Il-1rn, Il-6, Nlrp3, Tlr4*, and *Tnf*). Novel findings also indicated differences in sensome and epigenetic regulator genes, suggesting that high fiber can promote changes in a microglial-specific and epigenetic-dependent manner. Since *Il-1β, Tnf*, and *Il-6* gene expression was strongly decreased by diet, we correlated these genes with corresponding concentrations of butyrate and other SCFAs in the mice. For *Il-1β* and *Tnf* (trend for *Il-6*), higher butyrate correlated with lower pro-inflammatory gene expression. The mechanisms behind such an association are unclear, but many possible explanations exist, including regulation of FFARs on peripheral immune cells signaling to the brain or differential activation of the vagus nerve (56). It is important to note that not all effects seen by administration of butyrate were recapitulated by high fiber diet, so it will be important in future studies to determine the precise mechanisms of how high fiber diet affect microglial activation. For example, other SCFAs demonstrate anti-inflammatory properties (57) and high fiber diets can change other aspects of metabolism through variations in the gut microbiota leading to decreased inflammation (58), so these will be important avenues to explore.

Butyrate and other dietary factors can affect DNA methylation within various peripheral tissues (59). Our findings do not indicate any changes to DNA methylation in the hippocampus in response to fiber feeding. These effects paralleled the lack of differences in inflammatory gene expression within the hippocampus. These findings strengthen the idea that the anti-inflammatory changes seen with diet are likely microglial specific. It will be important to confirm these results in the future, along with modifications of histones and DNA hydroxymethylation that are known to be affected in *Il-1β* (60, 61). Furthermore, it is exciting that newly discovered histone modifications such as histone propionylation and butyrylation could have the potential to be regulated by dietary fiber (62).

A high fiber diet was not able to effectively diminish the exaggerated inflammatory responses to immune challenge in aged mice in the same way that was demonstrated with NaB treatment. This may be due to route of administration, as SCFAs from fiber feeding are metabolized by colonocytes within the gut. We were unable to compare differences between the amount of butyrate that may have been observed in the circulation and brain during high fiber feeding compared to a NaB injection. But this will be an important future step to determine how much butyrate from diet can be detected directly in the brain or if butyrate’s mechanisms of action on neuroinflammation are predominantly indirect (16). However, it is interesting that there was an interaction of age × diet in one of the CpG sites analyzed for *Il-1β* DNA methylation, in that aged mice had less DNA methylation than both adult groups, but adult mice with the high fiber diet had increased DNA methylation compared to adult mice with the low fiber diet. This may indicate that DNA methylation is more dynamic and responsive to a dietary intervention (63), but the mechanism of this is unclear. Using a lower dose of LPS or a live infection more accurately mimicking a typical immune response in humans may be necessary to see more nuanced differences and understand the epigenetic mechanisms involved in the inflammatory response of aged individuals.

In conclusion, data thus far support a role of high fermentable fiber in altering the microbiome and potentially immune infiltration/gut permeability, which supports beneficial microglial function in aged mice. Our results suggest that the effects of butyrate and potentially butyrate through high fiber feeding are due to a combination of mechanisms, including epigenetic regulation, metabolic regulation, and GPCR signaling. More work needs to be performed to examine the mode of action of butyrate-producing diets in the brain and whether or not these results relate to the beneficial effects seen with NaB. The characteristic that butyrate is synthesized endogenously *via* fermentation of non-digestible fibers by bacteria in the colon makes the prevention or at least suppression of neuroinflammatory conditions such as aging possible (16). High fiber supplementation in the elderly therefore may be a non-invasive strategy to increase butyrate levels and support an environment with decreased chronic neuroinflammation.

## Ethics Statement

All studies were carried out in accordance with United States National Institutes of Health guidelines and approved by the University of Illinois Institutional Animal Care and Use Committee.

## Author Contributions

SM, ML, JA, JW, and RJ contributed conception of the study. SM, JA, and ML contributed design of the study. SM, ML, and JA contributed significant intellectual content in order to perform the experiments of the study. SM, ML, JA, and LM performed the actual experiments. SM performed the statistical analysis and wrote the first draft of the manuscript. JA wrote sections of the manuscript. All the authors contributed to manuscript revision, read, and approved the submitted version. RJ was responsible for the final approval of the submitted version.

## Conflict of Interest Statement

The authors declare that the research was conducted in the absence of any commercial or financial relationships that could be construed as a potential conflict of interest.

## Supplementary Material

Figure S1 α-diversity rarefaction plots as determined by Chao1. No differences in α-diversity were observed as a result of age (blue: adult mice red: aged mice; shown above) or fiber feeding (data not shown).

Figure S2 β-diversity analysis (weighted Unifrac) reveals differences in gut microbiota community composition between high fiber-fed (green) and low fiber-fed (orange) mice after 4 weeks of feeding.

Figure S3 Hippocampal **(A)** Il-1β, **(B)** Tnf, **(C)** Il-6 gene expression and DNA methylation of the Il-1β promoter at **(D)** CpG1 and **(E)** CpG2 in adult and aged mice fed low or high fiber diets 4 h post-lipopolysaccharide (LPS) injection. Data are presented as mean ± SEM (*n* = 6–8). **(F)** Serum LPS binding protein (LBP) measured in adult and aged mice fed low or high fiber diets 4 h post-LPS injection. Data are presented as mean ± SEM (*n* = 7–9).

Figure S4 Gene expression of **(A)** colon and **(B)** lymph nodes from aged mice fed either a low or high fiber diet 4 h post-lipopolysaccharide (LPS) injection. Data are presented as mean ± SEM (*n* = 5–7). * indicates significance at *p* < 0.05 and ^#^ indicates significance at *p* < 0.1.
