# Supplementary figures and images for: Butyrate and Dietary Soluble Fiber Improve Neuroinflammation Associated With Aging in Mice
Source: Front Immunol. 2018 Aug 14;9:1832. doi: 10.3389/fimmu.2018.01832 (PMC6102557; doi:10.3389/fimmu.2018.01832)

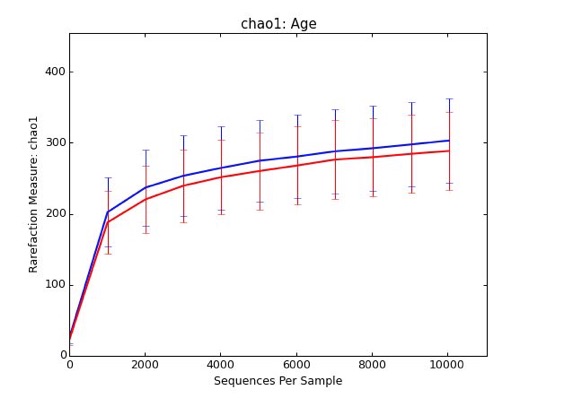

Supplement: Figure S1 — α-diversity rarefaction plots as determined by Chao1. No differences in α-diversity were observed as a result of age (blue: adult mice red: aged mice; shown above) or fiber feeding (data not shown). [file Image_1.JPEG]

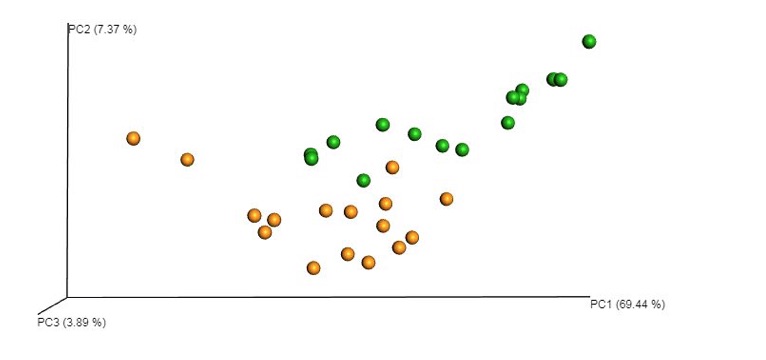

Supplement: Figure S2 — β-diversity analysis (weighted Unifrac) reveals differences in gut microbiota community composition between high fiber-fed (green) and low fiber-fed (orange) mice after 4 weeks of feeding. [file Image_2.JPEG]

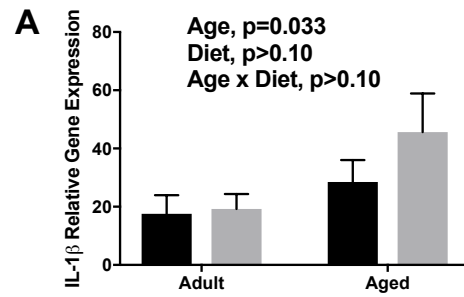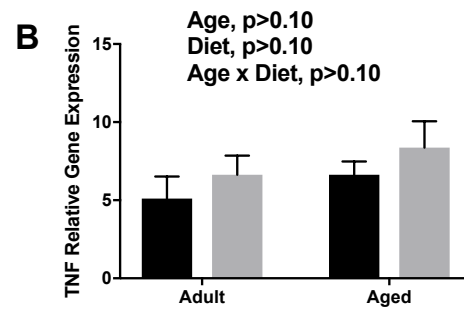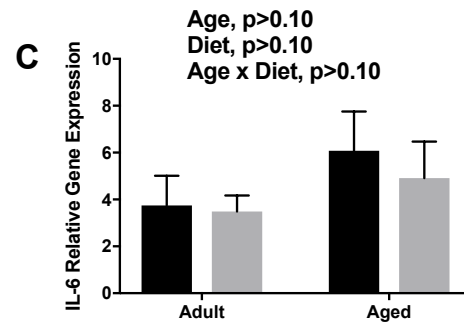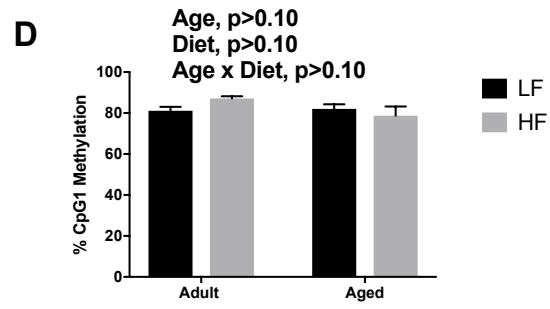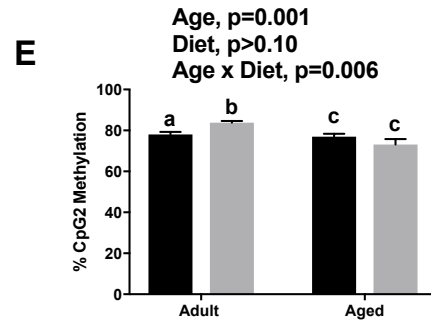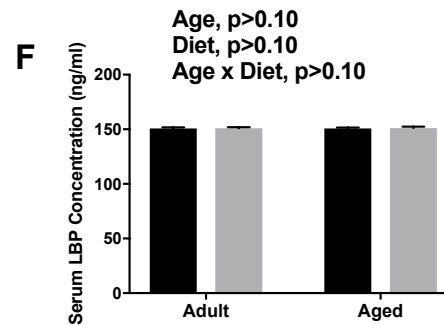

Supplement: Figure S3 — Hippocampal (A) Il-1β, (B) Tnf, (C) Il-6 gene expression and DNA methylation of the Il-1β promoter at (D) CpG1 and (E) CpG2 in adult and aged mice fed low or high fiber diets 4 h post-lipopolysaccharide (LPS) injection. Data are presented as mean ± SEM (n = 6–8). (F) Serum LPS binding protein (LBP) measured in adult and aged mice fed low or high fiber diets 4 h post-LPS injection. Data are presented as mean ± SEM (n = 7–9). [file Image_3.pdf]

**A**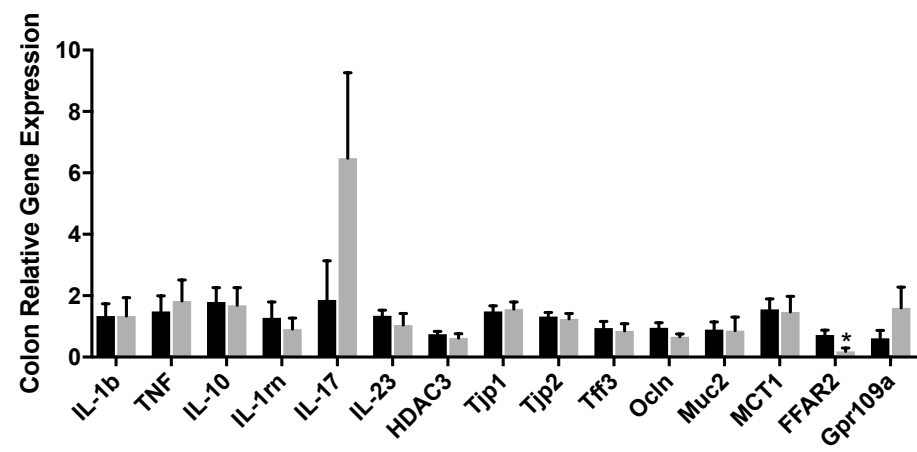**B**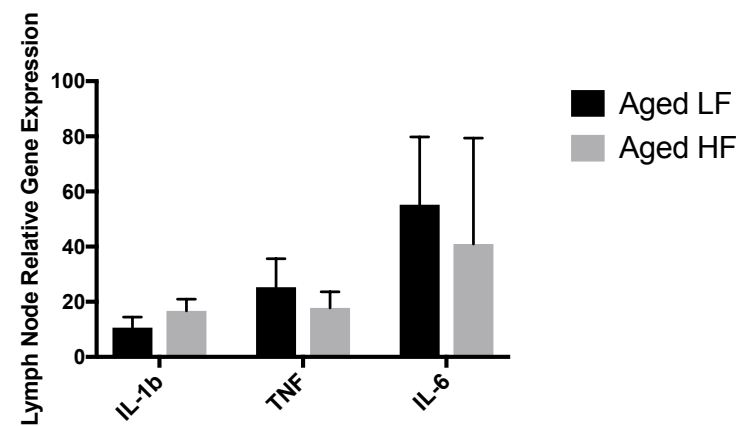

Supplement: Figure S4 — Gene expression of (A) colon and (B) lymph nodes from aged mice fed either a low or high fiber diet 4 h post-lipopolysaccharide (LPS) injection. Data are presented as mean ± SEM (n = 5–7). * indicates significance at p < 0.05 and # indicates significance at p < 0.1. [file Image_4.pdf]
