# Supplementary material for: Butyrate and Dietary Soluble Fiber Improve Neuroinflammation Associated With Aging in Mice
Source: Front Immunol. 2018 Aug 14;9:1832. doi: 10.3389/fimmu.2018.01832 (PMC6102557; doi:10.3389/fimmu.2018.01832)
Supplement: Supplementary file 5 [file Data_Sheet_1.docx]

| 1. Inflammatory and Regulators of Inflammatory Genes | | | | | | | |
| --- | --- | --- | --- | --- | --- | --- | --- |
|  | | | | | **p-values** | | |
| **Gene** | **SAL + SAL** | **NaB + SAL** | **SAL + LPS** | **NaB + LPS** | **NaB** | **LPS** | **NaB x LPS** |
| **Arg1** | **1.21±0.31** | **1.21±0.61** | **2.09±0.39** | **0.94±0.24** | **0.161** | **0.454** | **0.165** |
| **Casp1** | **0.95±0.19** | **1.21±0.25** | **0.77±0.14** | **0.52±0.10** | **0.98** | **0.029*** | **0.193** |
| **Cd68** | **0.91±0.13** | **1.08±0.20** | **0.56±0.08** | **0.37±0.04** | **0.929** | **0.0003*** | **0.188** |
| **Cx3cr1** | **0.97±0.17** | **0.97±0.13** | **0.41±0.06** | **0.23±0.02** | **0.378** | **<0.0001*** | **0.373** |
| **HIF-1α** | **0.88±0.02** | **1.18±0.25** | **1.52±0.41** | **0.93±0.23** | **0.720** | **0.624** | **0.262** |
| **Igf1** | **1.06±0.31** | **1.32±0.25** | **0.37±0.12** | **0.21±0.03** | **0.809** | **<0.0001*** | **0.269** |
| **Il-1β** | **1.22±0.65** | **1.13±0.18** | **3.29±0.37** | **1.83±0.21** | **0.045*** | **0.0005*** | **0.077** |
| **Il-1rn** | **0.99±0.35** | **0.81±0.11** | **19.03±3.82** | **14.91±3.25** | **0.436** | **<0.0001*** | **0.474** |
| **Il-10** | **1.10±0.35** | **0.80±0.16** | **5.19±0.77** | **2.21±0.56** | **0.004*** | **<0.0001*** | **0.017*** |
| **Il-6** | **1.23±0.59** | **0.86±0.12** | **1.32±0.25** | **1.01±0.20** | **0.277** | **0.709** | **0.934** |
| **Niarc1** | **1.16±0.55** | **1.09±0.20** | **6.67±1.52** | **5.41±1.09** | **0.529** | **<0.0001*** | **0.578** |
| **Nlrp3** | **0.84±0.07** | **1.59±0.52** | **1.05±0.25** | **0.80±0.21** | **0.548** | **0.484** | **0.234** |
| **Pycard** | **0.99±0.06** | **0.75±0.02** | **0.61±0.07** | **0.68±0.03** | **0.097** | **<0.0001*** | **0.004*** |
| **Socs1** | **1.01±0.25** | **1.74±0.28** | **1.39±0.17** | **1.40±0.26** | **0.143** | **0.947** | **0.148** |
| **Socs3** | **0.98±0.23** | **1.82±0.55** | **3.86±0.95** | **3.20±0.72** | **0.914** | **0.011*** | **0.361** |
| **Stat3** | **0.87±0.06** | **0.92±0.11** | **2.69±0.32** | **2.10±0.28** | **0.345** | **<0.0001*** | **0.264** |
| **Tlr2** | **0.94±0.15** | **1.13±0.18** | **1.78±0.38** | **1.71±0.41** | **0.877** | **0.062** | **0.730** |
| **Tlr4** | **1.02±0.14** | **1.01±0.16** | **0.47±0.06** | **0.36±0.04** | **0.599** | **<0.0001*** | **0.656** |
| **Tlr7** | **0.86±0.04** | **0.92±0.13** | **1.32±0.16** | **1.00±0.12** | **0.444** | **0.128** | **0.275** |
| **Tlr8** | **0.94±0.18** | **1.01±0.17** | **1.35±0.21** | **0.89±0.12** | **0.323** | **0.447** | **0.177** |
| **Tnf** | **0.97±0.14** | **1.01±0.20** | **1.84±0.31** | **1.35±0.23** | **0.371** | **0.02*** | **0.306** |

| 1. Sensome Genes | | | | | | | |
| --- | --- | --- | --- | --- | --- | --- | --- |
|  |  |  |  |  | **p-values** | | |
| **Gene** | **SAL + SAL** | **NaB + SAL** | **SAL + LPS** | **NaB + LPS** | **NaB** | **LPS** | **NaB x LPS** |
| **Cd53** | **0.92±0.09** | **0.96±0.13** | **0.93±0.12** | **0.71±0.08** | **0.475** | **0.340** | **0.300** |
| **Gpr34** | **0.96±0.12** | **1.14±0.22** | **0.20±0.05** | **0.08±0.02** | **0.822** | **<0.0001*** | **0.338** |
| **P2ry12** | **1.18±0.49** | **0.88±0.09** | **0.31±0.06** | **0.17±0.02** | **0.312** | **0.0004*** | **0.69** |
| **P2ry13** | **0.93±0.12** | **0.90±0.12** | **0.43±0.05** | **0.28±0.02** | **0.299** | **<0.0001*** | **0.509** |
| **Siglech** | **0.94±0.13** | **1.07±0.18** | **0.53±0.08** | **0.32±0.04** | **0.781** | **<0.0001*** | **0.192** |
| **Tgfbr1** | **0.93±0.18** | **1.19±0.24** | **0.53±0.08** | **0.34±0.04** | **0.842** | **0.0003*** | **0.170** |
| **Tmem119** | **1.09±0.27** | **2.04±0.62** | **0.55±0.11** | **0.32±0.05** | **0.362** | **0.005*** | **0.133** |
| **Trem2** | **0.93±0.31** | **2.16±0.61** | **0.86±0.19** | **0.62±0.14** | **0.192** | **0.035*** | **0.053** |

| 1. Epigenetic Regulator Genes | | | | | | | |
| --- | --- | --- | --- | --- | --- | --- | --- |
|  |  |  |  |  | **p-values** | | |
| **Gene** | **SAL + SAL** | **NaB + SAL** | **SAL + LPS** | **NaB + LPS** | **NaB** | **LPS** | **NaB x LPS** |
| **Hdac2** | **0.97±0.21** | **1.27±0.25** | **0.99±0.18** | **0.71±0.13** | **0.958** | **0.226** | **0.195** |
| **Hdac3** | **0.91±0.13** | **1.00±0.16** | **0.76±0.10** | **0.58±0.08** | **0.725** | **0.031*** | **0.305** |
| **Hdac4** | **0.93±0.29** | **1.61±0.32** | **3.67±0.92** | **3.16±0.73** | **0.902** | **0.004*** | **0.404** |
| **Hdac5** | **0.99±0.26** | **1.82±0.41** | **0.54±0.12** | **0.32±0.05** | **0.247** | **0.0004*** | **0.0457*** |
| **Hdac6** | **0.97±0.27** | **1.76±0.38** | **1.87±0.40** | **1.19±0.24** | **0.883** | **0.673** | **0.059** |
| **Tet1** | **0.94±0.07** | **1.00±0.16** | **0.29±0.07** | **0.12±0.02** | **0.621** | **<0.0001*** | **0.313** |
| **Tet2** | **1.03±0.18** | **1.23±0.23** | **1.32±0.29** | **1.23±0.25** | **0.855** | **0.633** | **0.641** |
| **Tet3** | **1.03±0.27** | **2.42±0.69** | **0.81±0.19** | **0.57±0.14** | **0.167** | **0.013*** | **0.049*** |

**Supplementary Table 1.** Expression of **(A)** inflammatory, **(B)** sensome, and **(C)** epigenetic regulator genes in microglia collected at 4 hours after SAL/LPS i.p. injections in adult mice pre-treated with i.p. SAL/NaB. Data are presented as means ± SEM (n=7-10) and p-values for main effects of NaB and LPS as well as NaB x LPS interactions are also included.

| 1. Inflammatory and Regulators of Inflammatory Genes | | | | | | | |
| --- | --- | --- | --- | --- | --- | --- | --- |
|  | | | | | **p-values** | | |
| **Gene** | **SAL + SAL** | **NaB + SAL** | **SAL + LPS** | **NaB + LPS** | **NaB** | **LPS** | **NaB x LPS** |
| **Arg1** | **2.82±1.46** | **1.38±0.55** | **5.65±1.37** | **4.69±0.75** | **0.252** | **0.005*** | **0.812** |
| **Casp1** | **0.62±0.13** | **0.73±0.13** | **0.45±0.10** | **0.49±0.07** | **0.482** | **0.053** | **0.790** |
| **Cd68** | **0.74±0.15** | **0.93±0.16** | **0.47±0.09** | **0.44±0.05** | **0.508** | **0.0016*** | **0.364** |
| **Cx3cr1** | **0.51±0.09** | **0.58±0.07** | **0.18±0.02** | **0.18±0.02** | **0.582** | **<0.0001*** | **0.576** |
| **HIF-1α** | **0.76±0.17** | **0.95±0.19** | **2.14±0.57** | **2.09±0.40** | **0.850** | **0.0013*** | **0.744** |
| **Igf1** | **1.15±0.33** | **1.55±0.33** | **0.26±0.07** | **0.24±0.04** | **0.360** | **<0.0001*** | **0.304** |
| **Il-1β** | **1.77±0.40** | **1.93±0.31** | **5.24±0.52** | **3.54±0.30** | **0.050*** | **<0.0001*** | **0.018*** |
| **Il-1rn** | **2.81±0.50** | **3.07±0.36** | **38.12±3.75** | **27.25±2.91** | **0.036*** | **<0.0001*** | **0.028*** |
| **Il-10** | **1.42±0.39** | **2.23±0.59** | **12.67±3.90** | **7.28±1.44** | **0.239** | **<0.0001*** | **0.113** |
| **Il-6** | **0.70±0.12** | **0.81±0.12** | **2.32±0.39** | **1.96±0.33** | **0.659** | **<0.0001*** | **0.398** |
| **Niarc1** | **1.34±0.26** | **1.52±0.27** | **7.38±1.53** | **8.21±1.43** | **0.656** | **<0.0001*** | **0.771** |
| **Nlrp3** | **1.02±0.34** | **1.14±0.29** | **1.26±0.45** | **1.16±0.24** | **0.964** | **0.699** | **0.744** |
| **Pycard** | **0.99±0.06** | **0.75±0.02** | **0.61±0.07** | **0.68±0.03** | **0.097** | **<0.0001*** | **0.004*** |
| **Socs1** | **1.71±0.18** | **2.05±0.19** | **1.69±0.31** | **1.41±0.25** | **0.899** | **0.176** | **0.205** |
| **Socs3** | **1.16±0.30** | **1.21±0.22** | **5.53±1.57** | **4.21±0.85** | **0.488** | **0.0001*** | **0.460** |
| **Stat3** | **0.59±0.08** | **0.55±0.06** | **2.36±0.32** | **1.98±0.22** | **0.306** | **<0.0001*** | **0.411** |
| **Tlr2** | **0.90±0.16** | **1.09±0.16** | **3.05±0.66** | **2.41±0.35** | **0.566** | **<0.0001*** | **0.294** |
| **Tlr4** | **0.62±0.12** | **0.66±0.10** | **0.41±0.06** | **0.33±0.03** | **0.816** | **0.0014*** | **0.510** |
| **Tlr7** | **0.55±0.08** | **0.54±0.07** | **0.96±0.14** | **0.88±0.08** | **0.657** | **0.0002*** | **0.700** |
| **Tlr8** | **1.34±0.27** | **1.41±0.28** | **2.30±0.51** | **1.89±0.26** | **0.612** | **0.035*** | **0.486** |
| **Tnf** | **1.23±0.26** | **1.62±0.28** | **3.13±0.74** | **2.49±0.37** | **0.783** | **0.003*** | **0.257** |

| 1. Sensome Genes | | | | | | | |
| --- | --- | --- | --- | --- | --- | --- | --- |
|  |  |  |  |  | **p-values** | | |
| **Gene** | **SAL + SAL** | **NaB + SAL** | **SAL + LPS** | **NaB + LPS** | **NaB** | **LPS** | **NaB x LPS** |
| **Cd53** | **0.54±0.10** | **0.55±0.08** | **0.64±0.08** | **0.74±0.007** | **0.487** | **0.093** | **0.571** |
| **Gpr34** | **0.54±0.01** | **0.60±0.11** | **0.05±0.01** | **0.06±0.01** | **0.681** | **<0.0001*** | **0.727** |
| **P2ry12** | **0.48±0.07** | **0.52±0.07** | **0.12±0.01** | **0.11±0.01** | **0.706** | **0.0004*** | **0.629** |
| **P2ry13** | **0.48±0.09** | **0.50±0.06** | **0.25±0.04** | **0.24±0.02** | **0.938** | **<0.0001*** | **0.849** |
| **Siglech** | **0.56±0.12** | **0.55±0.09** | **0.28±0.05** | **0.28±0.04** | **0.946** | **0.0006*** | **0.957** |
| **Tgfbr1** | **0.61±0.13** | **0.73±0.13** | **0.27±0.06** | **0.31±0.04** | **0.439** | **0.0002*** | **0.679** |
| **Tmem119** | **1.06±0.35** | **1.36±0.40** | **0.27±0.09** | **0.31±0.08** | **0.506** | **0.0006*** | **0.613** |
| **Trem2** | **1.52±0.48** | **2.07±0.49** | **0.68±0.24** | **0.68±0.14** | **0.449** | **0.0025*** | **0.439** |

| 1. Epigenetic Regulator Genes | | | | | | | |
| --- | --- | --- | --- | --- | --- | --- | --- |
|  |  |  |  |  | **p-values** | | |
| **Gene** | **SAL + SAL** | **NaB + SAL** | **SAL + LPS** | **NaB + LPS** | **NaB** | **LPS** | **NaB x LPS** |
| **Hdac2** | **0.78±0.17** | **0.84±0.16** | **0.61±0.11** | **0.62±0.07** | **0.802** | **0.138** | **0.842** |
| **Hdac3** | **0.59±0.11** | **0.65±0.10** | **0.53±0.08** | **0.52±0.06** | **0.750** | **0.278** | **0.677** |
| **Hdac4** | **1.09±0.26** | **1.11±0.22** | **2.32±0.52** | **3.00±0.57** | **0.432** | **0.0008*** | **0.452** |
| **Hdac5** | **1.14±0.32** | **1.67±0.33** | **0.28±0.07** | **0.27±0.05** | **0.257** | **<0.0001*** | **0.247** |
| **Hdac6** | **0.83±0.22** | **1.06±0.21** | **1.43±0.37** | **1.20±0.21** | **0.997** | **0.149** | **0.360** |
| **Tet1** | **0.48±0.08** | **0.51±0.07** | **0.07±0.01** | **0.07±0.01** | **0.747** | **<0.0001*** | **0.808** |
| **Tet2** | **0.86±0.18** | **0.89±0.16** | **1.34±0.28** | **1.40±0.27** | **0.843** | **0.040*** | **0.962** |
| **Tet3** | **1.16±0.35** | **1.65±0.43** | **0.71±0.20** | **0.65±0.09** | **0.466** | **0.014*** | **0.348** |

**Supplementary Table 2.** Expression of **(A)** inflammatory, **(B)** sensome, and **(C)** epigenetic regulator genes in microglia collected at 4 hours after SAL/LPS i.p. injections in aged mice pre-treated with i.p. SAL/NaB. Data are presented as means ± SEM (n=7-10) and p-values for main effects of NaB and LPS as well as NaB x LPS interactions are also included.

| 1. Inflammatory and Regulators of Inflammatory Genes | | | | | | | |
| --- | --- | --- | --- | --- | --- | --- | --- |
|  | | | | | **p-values** | | |
| **Gene** | **Adult LF** | **Adult HF** | **Aged LF** | **Aged HF** | **Age** | **Diet** | **Age x Diet** |
| **Casp1** | **0.89±0.11** | **1.10±0.13** | **0.98±0.11** | **0.84±0.09** | **0.415** | **0.744** | **0.127** |
| **Cd68** | **1.40±0.13** | **1.70±0.14** | **2.03±0.19** | **1.68±0.20** | **0.075** | **0.882** | **0.058** |
| **Cx3cr1** | **0.53±0.06** | **0.80±0.09** | **0.59±0.06** | **0.54±0.06** | **0.166** | **0.131** | **0.025*** |
| **HIF-1α** | **3.40±0.71** | **3.07±0.41** | **3.00±0.932** | **2.96±0.36** | **0.697** | **0.780** | **0.831** |
| **Ido1** | **1.95±0.28** | **1.78±0.28** | **2.36±0.50** | **2.37±0.42** | **0.191** | **0.839** | **0.813** |
| **Igf1** | **0.45±0.17** | **0.40±0.12** | **2.08±0.77** | **1.36±0.75** | **0.018*** | **0.468** | **0.522** |
| **Il-1β** | **7.17±1.04** | **8.01±0.80** | **12.41±1.07** | **14.55±2.10** | **<0.0001*** | **0.252** | **0.614** |
| **Il-1rn** | **10.38±1.94** | **8.31±1.48** | **15.19±3.22** | **18.89±3.59** | **0.005*** | **0.761** | **0.280** |
| **Il-10** | **7.42±1.88** | **5.46±0.72** | **9.64±1.93** | **9.38±1.67** | **0.070** | **0.507** | **0.609** |
| **Il-6** | **3.00±0.40** | **3.24±0.40** | **2.77±0.30** | **3.60±0.33** | **0.858** | **0.150** | **0.423** |
| **Niarc1** | **11.79±1.88** | **15.09±2.05** | **11.96±0.88** | **13.03±2.22** | **0.604** | **0.232** | **0.540** |
| **Nlrp3** | **2.56±0.37** | **2.87±0.36** | **2.45±0.27** | **2.65±0.45** | **0.649** | **0.485** | **0.887** |
| **Pycard** | **2.14±0.25** | **2.62±0.34** | **2.31±0.19** | **2.26±0.22** | **0.704** | **0.401** | **0.314** |
| **Socs1** | **1.13±0.05** | **1.02±0.07** | **0.97±0.05** | **0.99±0.08** | **0.163** | **0.474** | **0.280** |
| **Socs3** | **8.86±0.97** | **8.37±0.67** | **11.08±1.11** | **9.33±1.48** | **0.153** | **0.312** | **0.566** |
| **Stat3** | **4.08±0.50** | **4.32±0.53** | **3.62±0.34** | **3.06±0.41** | **0.064** | **0.722** | **0.385** |
| **Tlr2** | **3.03±0.51** | **2.58±0.32** | **3.17±0.29** | **3.44±0.56** | **0.250** | **0.832** | **0.399** |
| **Tlr4** | **0.72±0.07** | **1.00±0.14** | **0.80±0.06** | **0.77±0.06** | **0.439** | **0.171** | **0.097** |
| **Tlr7** | **2.35±0.27** | **3.24±0.50** | **1.87±0.19** | **1.67±0.16** | **0.002*** | **0.280** | **0.091** |
| **Tlr8** | **3.51±0.59** | **4.56±0.97** | **6.07±1.21** | **4.36±0.71** | **0.208** | **0.723** | **0.142** |
| **Tnf** | **4.77±0.64** | **6.65±0.79** | **7.02±0.75** | **7.66±0.81** | **0.034*** | **0.100** | **0.411** |

| 1. Sensome Genes | | | | | | | |
| --- | --- | --- | --- | --- | --- | --- | --- |
|  |  |  |  |  | **p-values** | | |
| **Gene** | **Adult LF** | **Adult HF** | **Aged LF** | **Aged HF** | **Age** | **Diet** | **Age x Diet** |
| **Cd53** | **1.83±0.27** | **3.19±0.87** | **0.91±0.17** | **2.67±0.73** | **0.297** | **0.028*** | **0.778** |
| **Gpr34** | **0.23±0.04** | **0.40±0.08** | **0.20±0.03** | **0.21±0.06** | **0.064** | **0.119** | **0.167** |
| **P2ry12** | **0.45±0.06** | **0.71±0.12** | **0.41±0.05** | **0.33±0.06** | **0.011*** | **0.263** | **0.047*** |
| **P2ry13** | **1.03±0.09** | **1.61±0.23** | **0.97±0.09** | **0.82±0.07** | **0.004*** | **0.136** | **0.013*** |
| **Siglech** | **0.71±0.07** | **1.04±0.15** | **0.67±0.05** | **0.59±0.05** | **0.010*** | **0.199** | **0.035*** |
| **Tgfbr1** | **1.04±0.12** | **1.80±0.36** | **1.02±0.14** | **0.96±0.15** | **0.054** | **0.120** | **0.065** |
| **Tmem119** | **1.00±0.17** | **2.36±0.49** | **1.28±0.25** | **1.34±0.32** | **0.271** | **0.036*** | **0.056** |
| **Trem2** | **1.73±0.19** | **2.66±0.33** | **2.03±1.34** | **1.46±0.25** | **0.123** | **0.527** | **0.011*** |

| 1. Epigenetic Regulator Genes | | | | | | | |
| --- | --- | --- | --- | --- | --- | --- | --- |
|  |  |  |  |  | **p-values** | | |
| **Gene** | **Adult LF** | **Adult HF** | **Aged LF** | **Aged HF** | **Age** | **Diet** | **Age x Diet** |
| **Dnmt3a** | **0.74±0.08** | **0.94±0.12** | **0.93±0.10** | **0.86±0.09** | **0.577** | **0.509** | **0.185** |
| **Dnmt3b** | **1.80±0.12** | **2.40±0.36** | **1.76±0.14** | **1.74±0.20** | **0.142** | **0.212** | **0.187** |
| **Gadd45b** | **3.83±0.62** | **4.75±0.57** | **6.27±0.68** | **7.25±1.03** | **0.0012*** | **0.006*** | **0.033*** |
| **Hdac1** | **0.90±0.03** | **0.81±0.04** | **0.75±0.04** | **0.78±0.04** | **0.012*** | **0.375** | **0.101** |
| **Hdac2** | **2.15±0.40** | **2.45±0.33** | **2.46±0.60** | **1.75±0.54** | **0.680** | **0.678** | **0.297** |
| **Hdac3** | **1.33±0.14** | **1.61±0.23** | **1.34±0.10** | **1.16±0.10** | **0.162** | **0.776** | **0.149** |
| **Hdac4** | **7.70±1.19** | **8.46±1.38** | **5.70±0.90** | **5.22±1.52** | **0.043*** | **0.913** | **0.624** |
| **Hdac5** | **0.45±0.08** | **1.33±0.30** | **0.61±0.12** | **0.70±0.18** | **0.236** | **0.015*** | **0.045*** |
| **Hdac6** | **3.44±0.45** | **3.79±0.80** | **3.38±0.76** | **3.33±0.94** | **0.735** | **0.841** | **0.800** |
| **Mecp2** | **1.42±0.16** | **1.76±0.20** | **1.35±0.11** | **1.22±0.12** | **0.052** | **0.498** | **0.140** |
| **Tet1** | **0.30±0.09** | **0.65±0.12** | **0.34±0.06** | **0.47±0.12** | **0.512** | **0.023*** | **0.279** |
| **Tet2** | **2.74±0.37** | **3.21±0.45** | **2.24±0.30** | **2.18±0.38** | **0.050*** | **0.589** | **0.492** |
| **Tet3** | **5.85±1.77** | **6.35±2.44** | **1.20±0.35** | **5.67±1.95** | **0.250** | **0.284** | **0.390** |

**Supplementary Table 3.** Expression of **(A)** inflammatory, **(B)** sensome, and **(C)** epigenetic regulator genes in microglia collected at 4 hours after SAL/LPS i.p. injections in aged mice pre-treated with i.p. SAL/NaB. Data are presented as means ± SEM (n=7-10) and p-values for main effects of NaB and LPS as well as NaB x LPS interactions are also included.
